# Development of novel 9-*O*-substituted-13-octylberberine derivatives as potential anti-hepatocellular carcinoma agents

**DOI:** 10.1080/14756366.2022.2118268

**Published:** 2022-09-06

**Authors:** Jichao Chen, Yiping Duan, Xiaoxuan Yu, Jiarou Zhong, Jing Bai, Nian-Guang Li, Zheying Zhu, Jinyi Xu

**Affiliations:** a School of Pharmacy, Nanjing University of Chinese Medicine, Nanjing, China; b State Key Laboratory of Natural Medicines and Department of Medicinal Chemistry, China Pharmaceutical University, Nanjing, China; c School of Medicine, Holistic Integrative Medicine, Nanjing University of Chinese Medicine, Nanjing, China; d School of Chemistry and Life Sciences, Suzhou University of Science and Technology, Suzhou, China; e Division of Molecular Therapeutics & Formulation, School of Pharmacy, The University of Nottingham, University Park Campus, Nottingham, UK

**Keywords:** Hepatocellular carcinoma, natural product, berberine, lipid-water partition coefficient, mitochondrial dysfunction

## Abstract

A series of novel 9-*O*-substituted-13-octylberberine derivatives were designed, synthesised and evaluated for their anti-hepatocellular carcinoma (HCC) activities. Compound **6k** showed the strongest activity against three human hepatoma cells including HepG2, Sk-Hep-1 and Huh-7 cells with IC_50_ values from 0.62 to 1.69 μM, which were much superior to berberine (IC_50_ >50 μM). More importantly, **6k** exhibited lower cytotoxicity against normal hepatocytes L-02 with good lipid-water partition properties. The mechanism studies revealed that **6k** caused G2/M phase arrest of the cell cycle, stabilised G-quadruplex DNA, and induced apoptosis via a mitochondrial apoptotic pathway. Finally, the *in vivo* anti-HCC activity of **6k** was validated in the H22 liver cancer xenograft mouse model. Collectively, the current study would provide a new insight into the discovery of novel, safe and effective anti-HCC agents.

## 1. Introduction

Liver cancer is the seventh most common cancer and the third leading cause of cancer-related death around the world, with an estimation of 905677 new cases and 830180 new deaths in 2020.^1^ Liver cancer patients are increasingly diagnosed per year, with an estimated incidence of >1 million cases by 2025.^2^ Hepatocellular carcinoma (HCC) constitutes over 80% of all liver cancers, possessing both high metastatic spread and extremely poor prognosis.^3^ Despite the substantial advances in drug discovery and development, the current treatment options for HCC are still limited. Surgical resection, local ablation and liver transplantation are applied with curative intention at the early stage of HCC,^4^^,^^5^ while most patients do not present with symptoms until their tumours develop rapidly into an advanced, often incurable, stage. The molecular targeted drugs, such as sorafenib and regorafenib, are used to treat patients with advanced HCC, however, they are associated with deleterious clinical side effects and high drug resistances.^6–8^ Hence, the development of novel, safe and effective agents in HCC therapy is urgently required.

**Figure 1.**
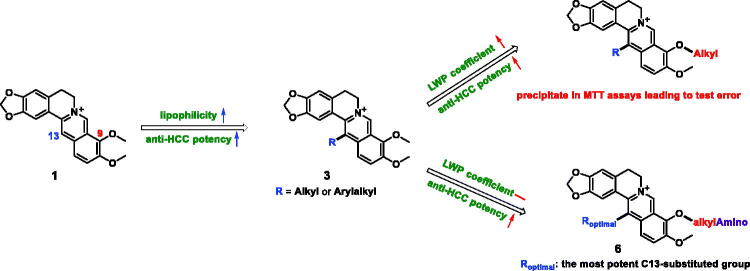
The design strategy of novel 9-*O*-13-disubstituted berberine derivatives.

Historically, natural products played a key role in drug discovery and development. There are over 80% of the approved small molecule antitumor agents deriving from natural products in the past 39 years from 1981 to 2019.^9^ Berberine (**1**, Figure 1), a natural isoquinoline alkaloid isolated from *Coptidis Rhizoma*, has shown a variety of pharmacological activities including anti-bacterial, anti-cancer, anti-inflammatory, anti-Alzheimer, anti-diabetic, anti-obesity, anti-viral, anti-hypertensive, anti-hyperlipidemic, *etc*.^10^^,^^11^ The antitumor effects of berberine have been demonstrated in various types of cancers, such as colorectal carcinoma, melanoma, gastric and breast cancers as well as HCC.^12^^,^^13^ Numerous studies revealed berberine exerted the anti-HCC activity via multiple mechanisms of action, including suppression of cell proliferation, migration and invasion, regulation of cell cycle, induction of cell apoptosis and autophagy, *etc*.^14^ Besides, several studies have shown that berberine could selectively inhibit hepatoma cell invasion and induce hepatoma cell apoptosis while eliciting less cytotoxicity in normal hepatocytes,^15^^,^^16^ and on the contrary, berberine could be combined with radiotherapy or chemotherapy drugs to neutralise their toxicity and enhance their therapeutic activities.^17–19^ Based on these attractive anticancer properties, berberine seems to be a promising candidate for the treatment of HCC, however, the poor absorption and moderate anticancer efficacy seriously hamper its further development and application.^20^

Berberine possesses poor lipid solubility, which leads to decreased effective concentration and minimal absorption in the gastrointestinal tract, thus exhibiting low antitumor effects.^21^ As a result, many efforts including derivatization have been devoted to enhancing berberine’s anticancer potential.^22^ Accumulating evidence has demonstrated that the introduction of an alkyl or arylalkyl group at the C9 or C13 position of berberine could improve the lipophilicity, making berberine easier to penetrate the cytomembrane, so as to enhance the antitumor efficacy.^22^ And our previous studies also confirmed that the introduction of alkyl chains of various lengths into the C9 position could effectively strengthen the anti-HCC activity of NO-donating berberine derivatives.^23^ To our knowledge, however, few studies were carried out to improve the antitumor potency on structural modification at both C9 and C13 positions of berberine.

In order to find potential anti-HCC agents, in our ongoing efforts on structural modification of berberine, firstly, several 9,13-dialkyl-substituted berberine derivatives were synthesised with high lipid-water partition (LWP) coefficients. While administrated against HepG2 cells, the derivatives were easy to precipitate in MTT assays due to low water solubility, leading to the test errors (data not provided). To overcome the above drawback, a series of novel 9-*O*-13-disubstituted berberine derivatives were designed to step by step (Figure 1). We first introduced various alkyl or arylalkyl groups into the C13 position (**3**) of berberine to obtain the most potent 13-alkyl or -arylalkyl berberine derivative by *in vitro* activity screening, then treating that as a lead compound, again introduced diverse aminoalkyl groups into its C9 position (**6**) to remain the LWP coefficient and further enhance the anti-HCC potency. Herein, we report the synthesis, *in vitro* and *in vivo* anti-HCC activity and mechanism studies for these novel derivatives.

## 2. Results and discussion

### 2.1. Chemistry

**Scheme 1.**
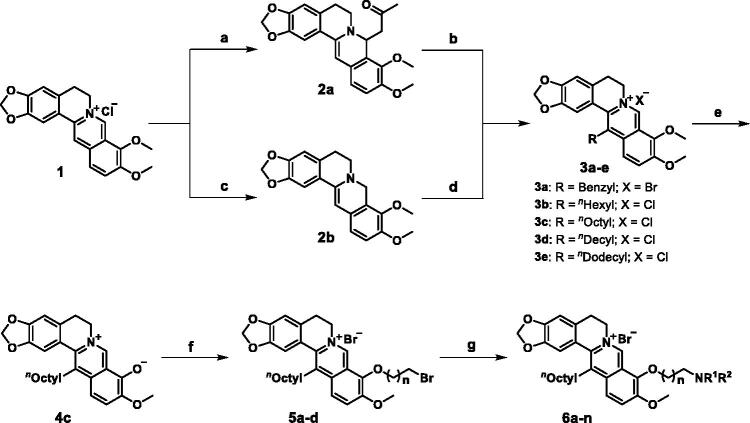
Synthetic routes of compounds **3a–e** and **6a–n**. Reagents and conditions: (a) 2 N NaOH, acetone, rt, 1.5 h; (b) BnBr, NaI, CH_3_CN, 85 °C, 8 h; (c) NaBH_4_, 5% NaOH, K_2_CO_3_, MeOH, rt, 2 h; (d) RCHO, AcOH, 80% EtOH, 90 °C, 5–8 h, then 1 N HCl, rt, 2 h; (e) 190 °C, 20–30 mmHg, 1 h; (f) dibromoalkanes, CH_3_CN, 85 °C, 5–10 h; (g) amines, Et_3_N, CH_3_CN, 85 °C, 8–12 h.

The synthesis of compounds **3a–e** and **6a–n** is illustrated in Scheme 1. The reaction of commercially available berberine (**1**) with acetone under the base produced compound **2a**, which was treated with benzyl bromide in the presence of NaI to give compound **3a**. On the other hand, **1** was reduced using NaBH_4_ under the base to obtain compound **2b**, followed by the reaction of diverse aldehydes in a mixed solvent of EtOH and AcOH to give compounds **3b**–**e**. Next, selective demethylation of **3c** through pyrolysis produced compound **4c**, which was directly reacted with different dibromoalkanes to form compounds **5a–d**, followed by treatment with various amines to afford compounds **6a–n**.

### 2.2. *In vitro* anti-HCC activity against HepG2 cells

Compounds **3a–e** were first synthesised and screened for their antiproliferative activity against HepG2 cells. As seen in Table 1, compound **3c** exhibited the strongest activity among the obtained C13-substituted berberine derivatives. Then taking **3c** as a precursor, compounds **6a–h** were prepared and evaluated. The results indicated compounds bearing terminal six-membered heterocycles such as morpholinyl (**6f**), piperidinyl (**6g**) and *N*-methylpiperazinyl (**6h**) groups displayed more potent activities than those with other substituted amino groups such as diethylamino (**6d**) and pyrrolidinyl (**6e**) groups (Table 1). Moreover, it was found that the activity increased by extending the alkyl chain length from C_3_ to C_6_ (e.g. **6d** *vs* **6a**, **6e** *vs* **6b**, **6f** *vs* **6c**, Table 1). Therefore, 9-*O*-substituted-13-octylberberine derivatives **6i–n** with longer alkyl chains and various six-membered heterocycles were further synthesised. The bioassay results indicated the anticancer activity gradually strengthened with the extension of the alkyl chain, however, the chain length had a little detrimental effect on the activity from C_8_ to C_10_ (e.g. **6i** *vs* **6l**, **6j** *vs* **6m**, **6k** *vs* **6n**, Table 1). While compounds bearing morpholinyl (e.g. **6i**), piperidinyl (e.g. **6j**) and *N*-methylpiperazinyl (e.g. **6k**) groups displayed comparably potent activity. Among all the derivatives, compound **6k** showed the strongest anti-HCC activity with an IC_50_ value of 0.76 μM, which was much superior to that of positive control cisplatin (IC_50_ 6.87 μM).

**Table 1.** Antiproliferative activity of berberine derivatives against HepG2 cells

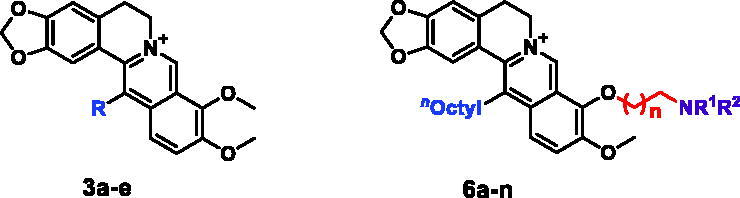				
Compd	*R*	*n*	-NR^1^R^2^	IC_50_^a^ (μM)
Berberine	/	/	/	> 50
**3a**	benzyl	/	/	> 50
**3b**	*^n^*hexyl	/	/	6.14 ± 0.57
**3c**	*^n^*octyl	/	/	4.12 ± 0.26
**3d**	*^n^*decyl	/	/	4.45 ± 0.34
**3e**	*^n^*dodecyl	/	/	4.24 ± 0.44
**6a**	*^n^*octyl	2	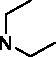	6.82 ± 0.51
**6b**	*^n^*octyl	2	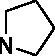	7.75 ± 0.83
**6c**	*^n^*octyl	2	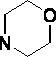	6.25 ± 0.56
**6d**	*^n^*octyl	5	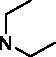	4.75 ± 0.24
**6e**	*^n^*octyl	5	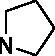	3.91 ± 0.36
**6f**	*^n^*octyl	5	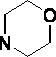	1.27 ± 0.25
**6g**	*^n^*octyl	5	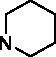	1.64 ± 0.13
**6h**	*^n^*octyl	5	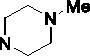	1.47 ± 0.19
**6i**	*^n^*octyl	7	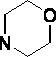	0.81 ± 0.08
**6j**	*^n^*octyl	7	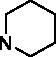	1.00 ± 0.10
**6k**	*^n^*octyl	7	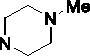	0.76 ± 0.06
**6l**	*^n^*octyl	9	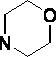	1.28 ± 0.11
**6m**	*^n^*octyl	9	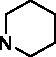	1.44 ± 0.13
**6n**	*^n^*octyl	9	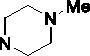	1.08 ± 0.10
Cisplatin	/	/	/	6.87 ± 0.31

^a^Concentration of the test compound that inhibits 50% of cell growth. Results are expressed as the mean ± SD (*n* = 3).

### 2.3. *In vitro* anti-HCC activity of 6k against a wide range of hepatoma cell lines

Compound **6k** was selected to examine the antiproliferative activity against other hepatoma cell lines including Sk-Hep-1, Huh-7 and Hep3B cells. As presented in Table 2, **6k** showed potent anti-HCC activities against the three cell lines with IC_50_ values of 1.69, 0.62 and 1.60 μM, respectively, much higher than berberine with IC_50_ values of >50 μM, even superior to positive control cisplatin with IC_50_ values of 6.52, 3.03 and 11.19 μM, respectively. More importantly, **6k** showed lower cytotoxicity towards normal hepatocytes L-02 compared to other hepatoma cell lines (Table 2). As a result, **6k** would be a potential anti-HCC agent and deserve further antitumor mechanism and *in vivo* studies.

**Table 2.** Antiproliferative activities of **6k** against other hepatic cell lines.

Compd	IC_50_^a^ (μM)			
	Sk-Hep-1	Huh-7	Hep3B	L-02
Berberine	>50	>50	>50	>50
**6k**	1.69 ± 0.07	0.62 ± 0.04	1.60 ± 0.11	7.68 ± 0.67
Cisplatin	6.52 ± 0.42	3.03 ± 0.63	11.19 ± 0.51	6.79 ± 0.46

^a^Concentration of the test compound that inhibits 50% of cell growth. Results are expressed as the mean ± SD (*n* = 3).

### 2.4. Effect of 6k on cell cycle progression

**Figure 2.**
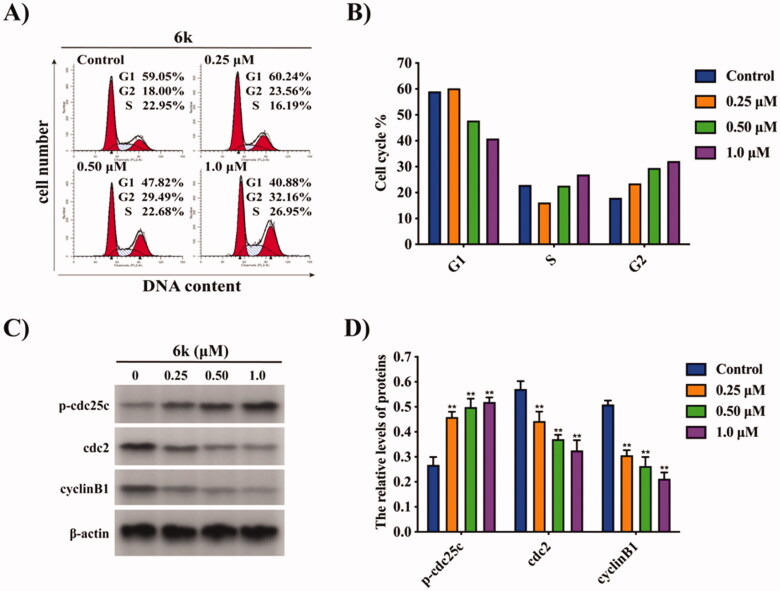
Effect of **6k** on cell cycle progression. HepG2 cells were treated with varying concentrations of **6k** for 72 h. (A) Cells were stained with PI and analysed by flow cytometry. (B) Histograms display the cell cycle distribution percentages. (C) Western blot analysis of G2/M-related proteins using *β*-actin as an internal control. (D) Histograms display the density ratios of *p*-cdc25c, cdc2 and cyclin B1 to *β*-actin. Data are expressed as the mean ± SD (*n* = 3). **p < 0.01 *vs*. control group.

To determine whether **6k** inhibited hepatoma cell proliferation by cell cycle regulation, the cell cycle distribution was analysed by flow cytometry after staining cellular DNA with propidium iodide (PI). As shown in Figure 2(A,B), **6k** caused cell cycle arrest at G2/M phase in a dose-dependent manner. Treatment of HepG2 cells with **6k** at concentrations from 0 to 1.0 μM increased the G2-phase cell percentages from 18.00% to 32.16%, while the total cell percentages of G1 and S phases decreased from 82.00% to 67.83% concomitantly. Then, the mechanism of **6k**-induced cell cycle arrest in HepG2 cells were further explored. The suppression of cell cycle progression from G2 to M phase in eukaryotic cells has been suggested to be associated with expressions of several regulatory proteins including cdc25c, cdc2 and cyclin B1^24^. As seen in Figure 2(C,D), **6k** significantly promoted cdc25c phosphorylation, inhibited cdc2 kinase activation and reduced cyclin B1 binding in concentration-dependent manners. These results indicated that **6k** blocked G2/M phase of the cell cycle possibly by regulating the expressions of *p*-cdc25c, cdc2 and cyclin B1.

### 2.5. Effect of 6k on G-quadruplex DNA

It is well known that G2 phase is the late stage of DNA synthesis and the preparation period for mitosis. During the progression of DNA replication, there are a number of G-quadruplex (G4) conformation present in oncogene (e.g. *c*-MYC) region, which are formed by diverse guanine-rich sequences, to maintain the stable existence of the replication action.^25^ G4 stabilisation by a small-molecule ligand could repress transcription of certain oncogenes and/or induce DNA damage at telomeres and oncogenes leading to replication defects and cancer cell death.^26^ Berberine has been reported to exert the anticancer activity related to its binding to G4 DNA.^27^

To investigate whether the anti-HCC activity of **6k** is involved with G4 DNA-binding effects, FRET-melting experiments were carried out with berberine as a reference. The fluorescent labelled oligomers FPu18T and F10T were employed as *c*-MYC G4 DNA and duplex DNA model, respectively. As shown in Table 3, **6k** could effectively stabilise G4 structure with a Δ*T*m(A) value of 13.8 °C, which was much higher than berberine (Δ*T*m: 1.2 °C). Whereas the melting temperature of F10T was not obviously elevated by **6k** with a Δ*T*m(B) value of 0.8 °C (Table 3), suggesting **6k** possessed lower binding affinity towards duplex DNA than G4 DNA. Besides, the G4 selectivity of **6k** was further examined by FRET-based competition assays using a nonfluorescent duplex DNA (ds26) as a competitor. The presence of excess ds26 didn’t obviously influence FPu18T stabilisation by **6k** with a Δ*T*m(C) value of 12.4 °C (Table 3), indicating the duplex DNA had negligible effect on the binding of **6k** to G4 DNA. Altogether, **6k** possessed high G4 DNA stabilising efficacy with better selectivity over duplex DNA.

**Table 3.** G4 DNA-binding effect of **6k** (°C).

Compd	Δ*T*_m_(A)^a^	Δ*T*_m_(B)^b^	Δ*T*_m_(C)^b^
Berberine	1.2	2.0	/
**6k**	13.8	0.8	12.4

^a^Δ*T*_m_ = *T*_m_(FPu18T + **6k**)-*T*_m_(FPu18T); ^b^Δ*T*_m_ = *T*_m_(F10T + **6k**)-*T*_m_(F10T).

### 2.6. Effect of 6k on cell apoptosis and mitochondrial membrane potential

**Figure 3.**
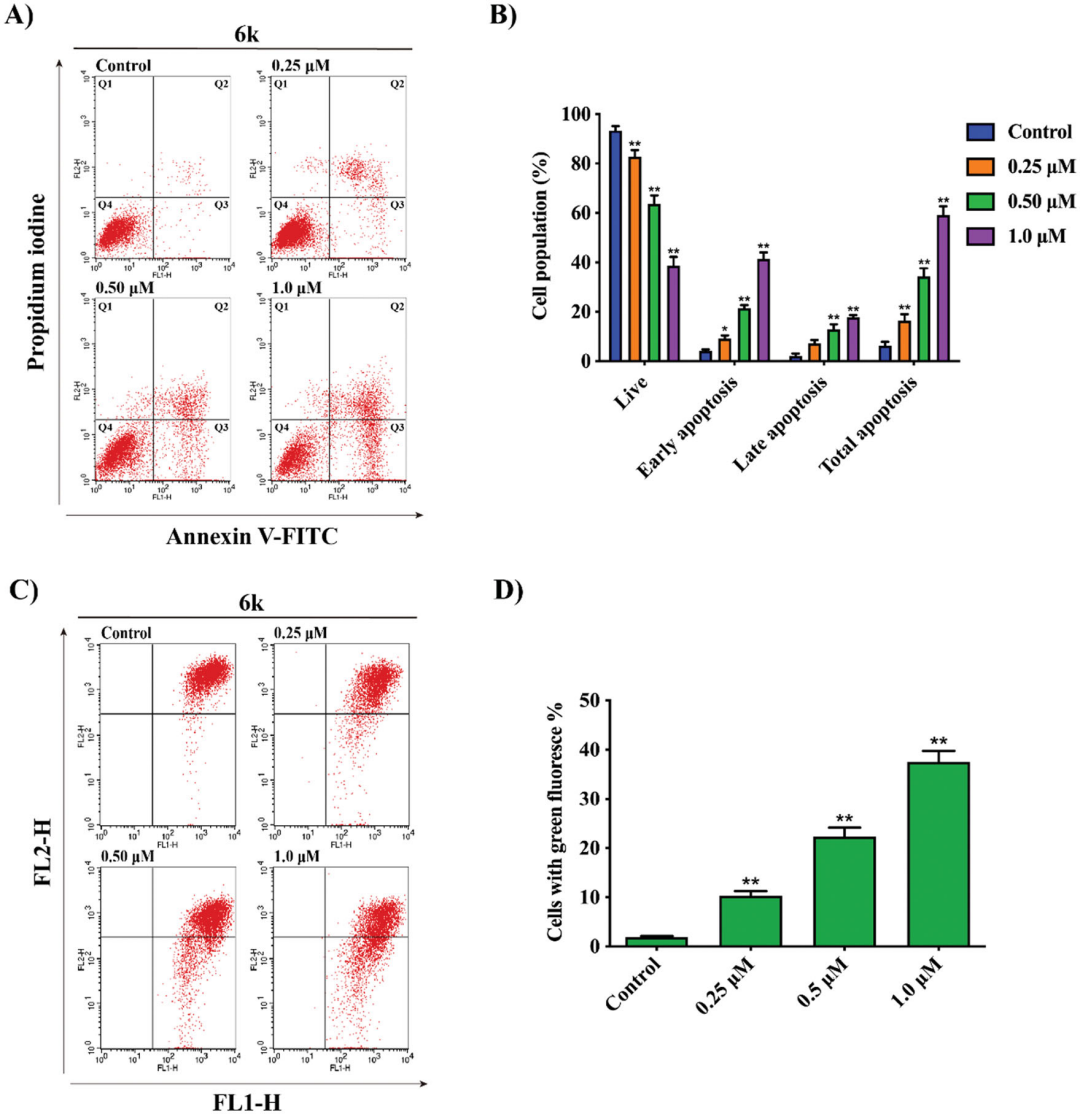
Effect of **6k** on cell apoptosis and MMP. HepG2 cells were treated with different concentrations of **6k** for 72 h. (A) Cells were stained with Annexin V-FITC/PI and analysed by flow cytometry. (B) Histograms display the percentages of cell distribution. (C) Cells were stained with JC-1 dye, followed by flow cytometry analysis. (D) Histograms display the percentages of cells with green fluorescence. Data are expressed as the mean ± SD (*n* = 3). *p < 0.05, **p < 0.01 *vs.* control group.

To verify whether cancer cell viability decreased by **6k** is associated with apoptosis, an Annexin V-FITC/PI binding assay was performed and the results were analysed by flow cytometry. As shown in Figure 3(A,B), **6k** markedly induced apoptosis in a dose-dependent manner. HepG2 cells were treated with **6k** at concentrations of 0.25, 0.50 and 1.0 μM for 72 h, which resulted in 16.42%, 34.29% and 59.17% total apoptotic cells (Q2 + Q3), as compared with 6.28% in the control group, indicating that **6k** exerted its anti-HCC effect possibly by inducing apoptosis.

It is well known that mitochondria play an important role in the process of apoptosis. Mitochondrial dysfunction triggers an alteration in mitochondrial structure, which can result in disruption of mitochondrial membrane potential (MMP) to initiate the apoptosis pathway^28^. In order to explore whether **6k** could induce mitochondrial dysfunction, the lipophilic mitochondrial probe JC-1 was employed to measure MMP. As shown in Figure 3(C,D), treatment with **6k** at concentrations from 0.25 to 1.0 μM for 72 h, the percentages of HepG2 cells with green fluorescence intensity correspondingly increased from 10.33% to 37.52%, as compared with 1.90% in the control group, suggesting that **6k** caused MMP collapse and mitochondrial dysfunction in the process of HepG2 apoptosis.

### 2.7. Effect of 6k on mitochondrial apoptotic pathway

**Figure 4.**
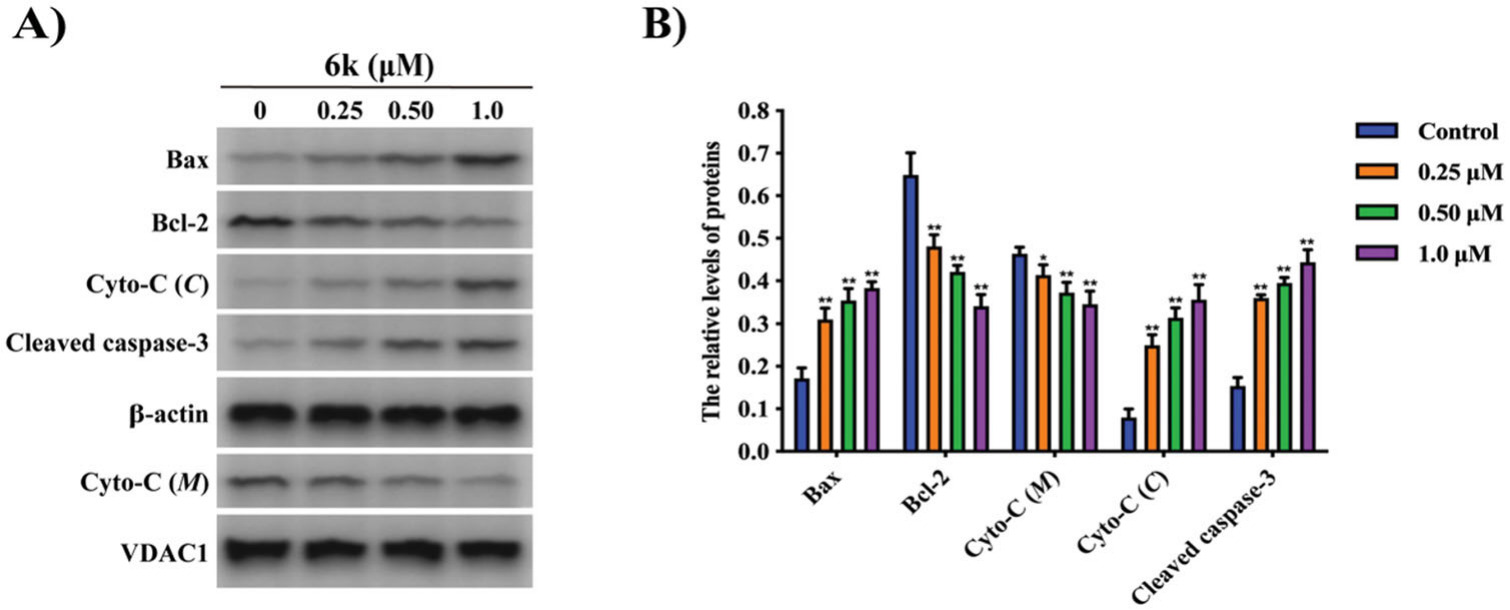
Effect of **6k** on the mitochondrial apoptotic pathway. HepG2 cells were treated with different concentrations of **6k** for 72 h. (A) The expression levels of apoptosis-related proteins were determined by Western Blotting using specific antibodies. *β*-Actin or VDAC1 was used as an internal control. (B) Histograms display the density ratios of Bax, Bcl-2, Cyto-C (C) and cleaved caspase-3 to *β*-actin and Cyto-C (*M*) to VDAC1. Cyto-C (*M*): mitochondrial cytochrome C; Cyto-C (C): cytosolic cytochrome C. Data are expressed as the mean ± SD (*n* = 3). *p < 0.05, **p < 0.01 *vs.* control group.

The above results demonstrated that apoptosis induced by **6k** was likely involved in a mitochondrial pathway. It has been found that when mitochondrial dysfunction occurs, cytochrome C releases from mitochondria into the cytoplasm across the membrane, which promotes the formation of a caspase activating complex that triggers the activation of downstream caspases^29^. Caspase-3 acts as a final executor in apoptosis and can be irreversibly activated through cleavage of procaspase-3^30^. Bcl-2, an anti-apoptotic protein, preserves mitochondrial structure and function.^31^ Bax, a dominant-negative inhibitor of Bcl-2, increases mitochondrial membrane permeability and promotes apoptosis.^31^ Thus, the expressions of Bax, Bcl-2, cytochrome C and caspase-3 were tested by western blot analysis. As shown in Figure 4(A,B), treatment with **6k** in HepG2 cells promoted cytochrome C release, activated caspase-3, downregulated Bcl-2 expression and upregulated Bax expression in concentration-dependent manners. The results indicated that **6k**-induced apoptosis could be mediated via a mitochondrial-dependent apoptotic pathway.

### 2.8. LWP property and aqueous solubility of 6k

In order to verify whether the physicochemical properties of berberine were improved, compounds **3c** and **6k** were selected to determine the LWP coefficient and aqueous solubility by the shake flask^32^ and HPLC^33^ methods, respectively. As expected in Table 4, compound **6k** could not only enhance the anti-HCC activity but also improve LWP property (Log D 3.87) compared to berberine (Log D −0.98). The water solubility of **6k** was 1.08 mg/mL, which was comparable to that of berberine (1.36 mg/mL).

**Table 4.** LWP coefficient and aqueous solubility of **6k** in PBS (*p*H 7.4).

Compd	*c*Log P^a^	Log D	Solubility (mg/mL)
Berberine	−1.54	−0.98	1.36
**3c**	2.66	2.41	1.12
**6k**	4.38	3.87	1.08

^a^Calculated LWP coefficient by ChemDraw 17.0.

### 2.9. *In vivo* anti-HCC activity of 6k

**Figure 5.**
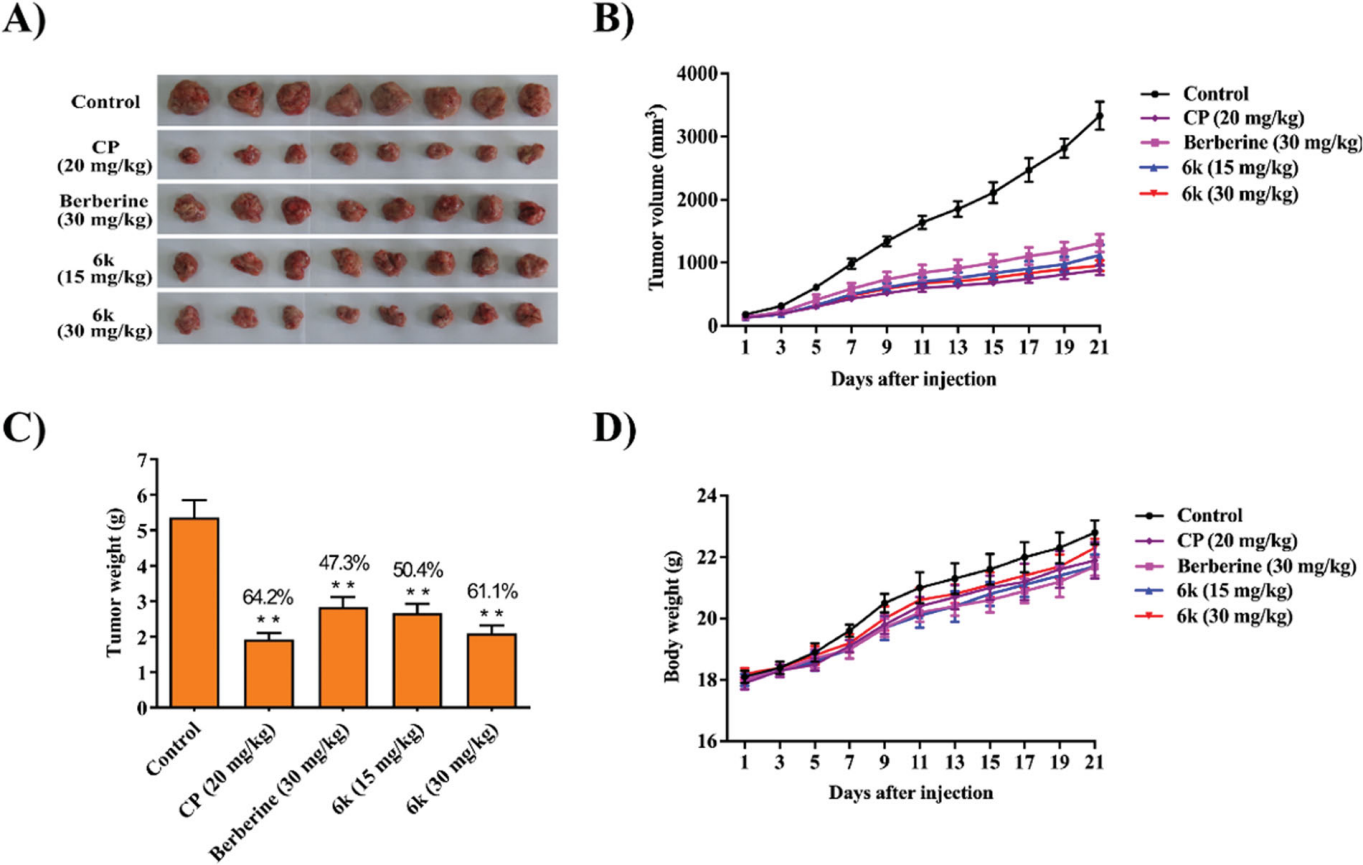
*In vivo* antitumor effect of **6k** in H22 liver cancer xenograft mouse model. After intravenously administering vehicle (10%DMF/2%Tween/88%saline, control), berberine (30 mg/kg), **6k** (15 or 30 mg/kg) and cyclophosphamide (CP, 20 mg/kg) once a day for 21 days, the mice were sacrificed and the tumours were weighed. (A) Images of tumours from mice after treatment of 21 days. (B) Tumour volume changes during treatment. (C) Weight of the excised tumours of each group. (D) Body weight changes of mice during treatment. Data are expressed as the mean ± SD (*n* = 8). **P < 0.01 *vs*. control group.

Encouraged by the above results, **6k** was selected to further test it is *in vivo* anti-HCC efficacy in mice liver cancer xenograft model established by subcutaneous inoculation of H22 cells into the right flank of mice. The tumour sizes and body weights of the mice were monitored and recorded every 2 days. All the mice were sacrificed after 3 weeks, the tumours were excised and weighed (Figure 5A). As illustrated in Figure 5(B,C), **6k** markedly inhibited the tumour growth (both volume and weight) in a dose-dependent manner. At a dose of 30 mg/kg, **6k** possessed a much higher tumour inhibitory rate (TIR 61.1%) than berberine (TIR 47.3%). Besides, **6k** didn’t cause significant loss in mice body weights during treatment (Figure 5D).

## 3. Conclusion

In summary, a series of novel 9-*O*-substituted-13-octylberberine derivatives were designed to step by step, prepared and their *in vitro* anti-HCC activities were initially evaluated. The results indicated that all the derivatives exhibited markedly improved antiproliferative activity compared to the parent compound berberine. Among them, compound **6k** showed the most potent activity (IC_50_ 0.62 ∼ 1.69 μM) against a wide range of human hepatoma cells while displaying lower cytotoxicity towards normal hepatocytes. The mechanism studies indicated that **6k** blocked G2/M phase of the cell cycle possibly by influencing the expressions of *p*-cdc25c, cdc2 and cyclin B1, stabilised G4 DNA with higher selectivity over duplex DNA, caused MMP loss and induced apoptosis through a mitochondrial apoptotic pathway. Besides, **6k** possessed good lipid-water partition property (log D 3.87) and aqueous solubility (1.08 mg/mL). Furthermore, **6k** effectively suppressed the tumour growth by 61.1% (*w/w*) in the H22 liver cancer xenograft mouse model, which was significantly superior to berberine (47.3%, *w*/*w*) at the same dose. Altogether, compound **6k** deserves to be further investigated as a potential anti-HCC agent for cancer therapy.

## 4. Experimental section

### 4.1. Chemistry

#### 4.1.1. General

Most chemicals and solvents were purchased from commercial sources. Further purification and drying by standard methods were employed when necessary. Melting points (m.p.) were taken on an XT-4 micro melting point apparatus and uncorrected. ^1^H NMR and ^13 ^C NMR spectra were recorded on Bruker-300 spectrometers in the indicated solvents (CDCl_3_ or DMSO-*d_6_*, TMS as internal standard): the values of the chemical shifts are expressed in δ values (ppm) and the coupling constants (J) in Hz. High-Resolution Mass measurement was performed on Agilent QTOF 6520 mass spectrometer with electron spray ionisation (ESI) as the ion source. The purity of all tested compounds was ≥95%, as estimated by HPLC analysis. Reactions were monitored by thin-layer chromatography (TLC) on 0.25 mm silica gel plates (GF254) and visualised under UV light. Flash column chromatography was carried out using commercially available silica gel (200–300 mesh) under pressure.

#### 4.1.2. General procedure for synthesis of compound 3

##### 4.1.2.1. Synthesis of compound 2

To a stirred 2 N NaOH (8 ml) solution of berberine **1** (1.86 g, 5 mmol) was dropwise added acetone (4 ml), the mixture was stirred at room temperature for 1.5 h. The mixture was then filtered and washed with 80% ethanol (15 ml) to provide the crude compound **2a** without further purification for next step directly.

To a stirred solution of berberine **1** (3.72 g, 10 mmol) and K_2_CO_3_ (4.14 g, 30 mmol) in MeOH (100 ml) was dropwise added a 5% NaOH (10 ml) solution containing NaBH_4_ (570 mg, 15 mmol), the mixture was stirred at room temperature for 2 h. The mixture was then filtered, and washed with water (30 ml) and 80% ethanol (30 ml) to provide the crude compound **2b** without further purification for next step directly.

##### 4.1.2.2. Synthesis of compound 3

To a solution of compound **2a** (1.18 g, 3 mmol) in acetonitrile (10 ml), sodium iodide (450 mg, 3 mmol) and benzyl bromide (1.1 ml, 9 mmol) were added. The reaction mixture was stirred at 85 °C for 8 h. The solvent was removed under reduced pressure, the residue was purified by silica gel column chromatography using dichloromethane/methanol as an eluent to give compound **3a**.

13-Benzyl-9,10-dimethoxy-5,6-dihydro-[1,3]dioxolo[4,5-g]isoquinolino[3,2-a]isoquinolin-7-ium bromide (**3a**) Yellow solid in 64% yield (972 mg). m.p. 243 − 245 °C. ^1^H NMR (300 MHz, DMSO-*d*_6_) *δ* 10.05 (s, 1H), 8.10 (d, *J* = 9.5 Hz, 1H), 7.78 (d, *J* = 9.5 Hz, 1H), 7.39 − 7.35 (m, 2H), 7.28 (t, *J* = 7.1 Hz, 1H), 7.18 − 7.16 (m, 3H), 6.96 (s, 1H), 6.08 (s, 2H), 4.88 (s, 2H), 4.75 (s, 2H), 4.12 (s, 3H), 4.02 (s, 3H), 3.18 − 3.14 (m, 2H). ^13 ^C NMR (75 MHz, DMSO-*d*_6_) *δ* 150.2, 149.2, 146.4, 145.5, 144.2, 139.2 137.1, 134.1, 132.7, 130.0, 129.1, 128.0, 126.8, 126.1, 121.7, 121.2, 120.0, 108.5, 108.1, 102.1, 62.1, 56.9 (2), 35.5, 27.3. HRMS (ESI) calculated for C_27_H_24_BrNO_4_ [M-Br]^+^ 426.1700, found 426.1702.

To a mixed solution of compound **2b** (2.02 g, 6 mmol) in 80% ethanol (16 ml) and AcOH (4 ml), different aldehydes (18 mmol) were added. The reaction mixture was stirred at 90 °C for 5–8 h. The solvent was removed under reduced pressure, the residue was then acidified with 1 N HCl (20 ml), and stirred at room temperature for 2 h. The solid was collected by filtration and then purified by silica gel column chromatography using dichloromethane/methanol as an eluent to give compounds **3b–e**.

13-Hexyl-9,10-dimethoxy-5,6-dihydro-[1,3]dioxolo[4,5-g]isoquinolino[3,2-a]isoquinolin-7-ium chloride (**3b**) Yellow solid in 58% yield (1.86 g). m.p. 183 − 185 °C. ^1^H NMR (300 MHz, CDCl_3_) *δ* 10.69 (s, 1H), 7.89 (d, *J* = 9.5 Hz, 1H), 7.84 (d, *J* = 9.5 Hz, 1H), 7.09 (s, 1H), 6.89 (s, 1H), 6.10 (s, 2H), 5.28 (s, 2H), 4.36 (s, 3H), 4.07 (s, 3H), 3.29 − 3.23 (m, 2H), 3.16 (s, 2H), 1.85 (s, 2H), 1.57 − 1.47 (m, 2H), 1.41 − 1.29 (m, 4H), 0.91 (t, *J* = 6.8 Hz, 3H). ^13 ^C NMR (75 MHz, CDCl_3_) *δ* 150.3, 149.6, 147.1, 145.8, 145.3, 135.9, 134.4, 133.7, 132.8, 125.6, 121.9, 120.3, 120.2, 108.9, 108.4, 102.1, 62.8, 57.5, 57.0, 31.11, 31.07, 29.8, 29.1, 28.6, 22.4, 13.9. HRMS (ESI) calculated for C_26_H_30_ClNO_4_ [M-Cl]^+^ 420.2169, found 420.2167.

9,10-Dimethoxy-13-octyl-5,6-dihydro-[1,3]dioxolo[4,5-g]isoquinolino[3,2-a]isoquinolin-7-ium chloride (**3c**) Yellow solid in 62% yield (1.80 g). m.p. 175 − 177 °C. ^1^H NMR (300 MHz, DMSO-*d*_6_) *δ* 9.90 (s, 1H), 8.20 (s, 2H), 7.29 (s, 1H), 7.17 (s, 1H), 6.19 (s, 2H), 4.79 (s, 2H), 4.09 (s, 6H), 3.09 − 3.06 (m, 2H), 1.77 − 1.70 (m, 2H), 1.39 − 1.33 (m, 2H), 1.28 − 1.23 (m, 10H), 0.85 (t, *J* = 6.6 Hz, 3H). ^13 ^C NMR (75 MHz, CDCl_3_) *δ* 150.5, 149.7, 147.3, 146.3, 145.8, 136.0, 134.3, 133.9, 133.0, 125.6, 122.1, 120.34, 120.28, 109.1, 108.6, 102.2, 63.1, 57.6, 57.1, 31.8, 31.2, 29.9, 29.6, 29.2, 29.1, 28.8, 22.6, 14.1. HRMS (ESI) calculated for C_28_H_34_ClNO_4_ [M-Cl]^+^ 448.2482, found 448.2483.

13-Decyl-9,10-dimethoxy-5,6-dihydro-[1,3]dioxolo[4,5-g]isoquinolino[3,2-a]isoquinolin-7-ium chloride (**3d**) Yellow solid in 48% yield (1.47 g). m.p. 187 − 189 °C. ^1^H NMR (300 MHz, CDCl_3_) *δ* 10.7 (s, 1H), 7.89 (d, *J* = 9.5 Hz, 1H), 7.84 (d, *J* = 9.5 Hz, 1H), 7.10 (s, 1H), 6.89 (s, 1H), 6.10 (s, 2H), 5.28 (s, 2H), 4.36 (s, 3H), 4.07 (s, 3H), 3.29 − 3.23 (m, 2H), 3.16 (t, *J* = 5.6 Hz, 2H), 1.90 − 1.78 (m, 2H), 1.56 − 1.47 (m, 2H), 1.41 − 1.25 (m, 12H), 0.90 − 0.86 (m, 3H). ^13 ^C NMR (75 MHz, CDCl_3_) *δ* 150.4, 149.6, 147.2, 146.0, 145.5, 135.9, 134.3, 133.8, 132.9, 125.5, 122.0, 120.31, 120.26, 109.0, 108.5, 102.1, 62.9, 57.5, 57.0, 31.8, 31.2, 29.9, 29.52, 29.48 (2), 29.2, 29.0, 28.6, 22.6, 14.1. HRMS (ESI) calculated for C_30_H_38_ClNO_4_ [M-Cl]^+^ 476.2795, found 476.2813.

13-Dodecyl-9,10-dimethoxy-5,6-dihydro-[1,3]dioxolo[4,5-g]isoquinolino[3,2-a]isoquinolin-7-ium chloride (**3e**) Yellow solid in 43% yield (1.39 g). m.p. 179 − 181 °C. ^1^H NMR (300 MHz, CDCl_3_) *δ* 10.46 (s, 1H), 7.87 (s, 2H), 7.04 (s, 1H), 6.84 (s, 1H), 6.05 (s, 2H), 5.17 (s, 2H), 4.23 (s, 3H), 4.02 (s, 3H), 3.24 − 3.19 (m, 2H), 3.11 (s, 2H), 1.79 (s, 2H), 1.49 − 1.40 (m, 2H), 1.30 − 1.16 (m, 16H), 0.83 − 0.79 (m, 3H). ^13 ^C NMR (75 MHz, CDCl_3_) *δ* 150.4, 149.7, 147.2, 146.1, 145.6, 136.0, 134.3, 133.9, 133.0, 125.6, 122.1, 120.3 (2), 109.0, 108.6, 102.1, 63.0, 57.6, 57.1, 31.9, 31.2, 29.9, 29.61, 29.59, 29.57, 29.5, 29.3, 29.1, 28.7, 22.7, 14.1. HRMS (ESI) calculated for C_32_H_42_ClNO_4_ [M-Cl]^+^ 504.3108, found 504.3118.

#### 4.1.3. General procedure for synthesis of compound 6

##### 4.1.3.1. Synthesis of compound 4c

Compound **3c** (1.70 g, 3.5 mmol) was heated at 190 °C in a vacuum oven under reduced pressure (20–30 mmHg) for 1 h to obtain the crude compound **4c** without further purification for the next step directly.

##### 4.1.3.2. Synthesis of compound 5

To a solution of compound **4c** (1.30 g, 3 mmol) in acetonitrile (10 ml), different dibromoalkanes (9 mmol) were added. The reaction mixture was stirred at 85 °C for 5–10 h. The solvent was removed under reduced pressure, the residue was purified by silica gel column chromatography using dichloromethane/methanol as an eluent to give compounds **5a–d**.

9–(3-Bromopropoxy)-10-methoxy-13-octyl-5,6-dihydro-[1,3]dioxolo[4,5-g]isoquinolino[3,2-a]isoquinolin-7-ium bromide (**5a**) Yellow solid in 67% yield (1.28 g). ^1^H NMR (300 MHz, CDCl_3_) *δ* 10.53 (s, 1H), 7.91 (d, *J* = 9.4 Hz, 1H), 7.86 (d, *J* = 9.4 Hz, 1H), 7.09 (s, 1H), 6.87 (s, 1H), 6.09 (s, 2H), 5.25 (s, 2H), 4.66 (t, *J* = 5.6 Hz, 2H), 4.07 (s, 3H), 3.83 (t, *J* = 5.9 Hz, 2H), 3.28 − 3.22 (m, 2H), 3.15 − 3.12 (m, 2H), 2.70 − 2.63 (m, 2H), 1.89 − 1.80 (m, 2H), 1.55 − 1.46 (m, 2H), 1.39 − 1.23 (m, 8H), 0.89 − 0.85 (m, 3H). MS (ESI) *m/z*: 554.13 [M-Br]^+^.

9-((6-Bromohexyl)oxy)-10-methoxy-13-octyl-5,6-dihydro-[1,3]dioxolo[4,5-g]isoquinolino[3,2-a]isoquinolin-7-ium bromide (**5b**) Yellow solid in 78% yield (1.59 g). ^1^H NMR (300 MHz, CDCl_3_) *δ* 10.17 (s, 1H), 7.88 (s, 2H), 7.04 (s, 1H), 6.82 (s, 1H), 6.04 (s, 2H), 5.14 (s, 2H), 4.43 (t, *J* = 6.8 Hz, 2H), 4.02 (s, 3H), 3.37 (t, *J* = 6.8 Hz, 2H), 3.23 − 3.18 (m, 2H), 3.12 (t, *J* = 5.6 Hz, 2H), 2.02 − 1.93 (m, 2H), 1.89 − 1.74 (m, 4H), 1.57 − 1.40 (m, 6H), 1.35 − 1.18 (m, 8H), 0.84 − 0.79 (m, 3H). MS (ESI) *m/z*: 596.26 [M-Br]^+^

9-((8-Bromooctyl)oxy)-10-methoxy-13-octyl-5,6-dihydro-[1,3]dioxolo[4,5-g]isoquinolino[3,2-a]isoquinolin-7-ium bromide (**5c**) Yellow solid in 72% yield (1.52 g). ^1^H NMR (300 MHz, CDCl_3_) *δ* 10.35 (s, 1H), 7.89 (d, *J* = 9.5 Hz, 1H), 7.84 (d, *J* = 9.5 Hz, 1H), 7.08 (s, 1H), 6.88 (s, 1H), 6.09 (s, 2H), 5.22 (s, 2H), 4.46 (t, *J* = 6.6 Hz, 2H), 4.05 (s, 3H), 3.52 (t, *J* = 6.6 Hz, 2H), 3.27 − 3.22 (m, 2H), 3.16 (s, 2H), 2.06 − 1.97 (m, 2H), 1.90 − 1.71 (m, 4H), 1.54 − 1.46 (m, 4H), 1.45 − 1.23 (m, 14H), 0.89 − 0.85 (m, 3H). MS (ESI) *m/z*: 624.31 [M-Br]^+^

9-((10-Bromodecyl)oxy)-10-methoxy-13-octyl-5,6-dihydro-[1,3]dioxolo[4,5-g]isoquinolino[3,2-a]isoquinolin-7-ium bromide (**5d**) Yellow solid in 68% yield (1.50 g). ^1^H NMR (300 MHz, CDCl_3_) *δ* 10.41 (s, 1H), 7.88 (d, *J* = 9.5 Hz, 1H), 7.83 (d, *J* = 9.5 Hz, 1H), 7.09 (s, 1H), 6.89 (s, 1H), 6.10 (s, 2H), 5.26 (s, 2H), 4.50 (t, *J* = 6.6 Hz, 2H), 4.05 (s, 3H), 3.53 (t, *J* = 6.7 Hz, 2H), 3.28 − 3.23 (m, 2H), 3.18 (s, 2H), 2.09 − 1.97 (m, 2H), 1.91 − 1.71 (m, 4H), 1.55 − 1.46 (m, 4H), 1.41 − 1.24 (m, 18H), 0.91 − 0.87 (m, 3H). MS (ESI) *m/z*: 652.28 [M-Br]^+^

##### 4.1.3.3. Synthesis of compound 6

To a solution of compound **5** (0.2 mmol) in acetonitrile (3 ml), different amines (0.6 mmol) and trimethylamine (83 μL, 0.6 mmol) were added. The reaction mixture was stirred at 85 °C for 8–12 h. The solvent was removed under reduced pressure, the residue was purified by silica gel column chromatography using dichloromethane/methanol as an eluent to give compounds **6a–n**.

9–(3-(Diethylamino)propoxy)-10-methoxy-13-octyl-5,6-dihydro-[1,3]dioxolo[4,5-g]isoquinolino[3,2-a]isoquinolin-7-ium bromide (**6a**). Yellow solid in 64% yield (80 mg). m.p. 142 − 144 °C. ^1^H NMR (300 MHz, CDCl_3_) *δ* 10.60 (s, 1H), 7.88 (d, *J* = 9.2 Hz, 1H), 7.83 (d, *J* = 9.4 Hz, 1H), 7.09 (s, 1H), 6.89 (s, 1H), 6.10 (s, 2H), 5.30 (s, 2H), 4.58 (t, *J* = 6.4 Hz, 2H), 4.06 (s, 3H), 3.28 − 3.23 (m, 2H), 3.16 (t, *J* = 5.5 Hz, 2H), 2.92 − 2.87 (m, 2H), 2.70 (q, *J* = 7.0 Hz, 4H), 2.34 − 2.25 (m, 2H), 1.90 − 1.81 (m, 2H), 1.55 − 1.46 (m, 2H), 1.41 − 1.21 (m, 8H), 1.11 (t, *J* = 7.1 Hz, 6H), 0.91 − 0.86 (m, 3H). ^13 ^C NMR (75 MHz, CDCl_3_) *δ* 150.0, 149.7, 147.2, 145.2, 144.5, 136.0, 134.4, 133.7, 132.8, 125.2, 121.8, 120.4, 120.2, 109.0, 108.4, 102.1, 71.7, 57.2, 57.0, 49.2, 46.8, 31.7, 31.1, 29.8, 29.4, 29.1, 28.9, 28.6, 25.0, 22.5, 14.0, 9.1. HRMS (ESI) calculated for C_34_H_47_BrN_2_O_4_ [M-Br]^+^ 547.3530, found 547.3539.

10-Methoxy-13-octyl-9–(3-(pyrrolidin-1-yl)propoxy)-5,6-dihydro-[1,3]dioxolo[4,5-g]isoquinolino[3,2-a]isoquinolin-7-ium bromide (**6b**) Yellow solid in 67% yield (84 mg). m.p. 135 − 137 °C. ^1^H NMR (300 MHz, CDCl_3_) *δ* 10.59 (s, 1H), 7.88 (d, *J* = 9.5 Hz, 1H), 7.83 (d, *J* = 9.4 Hz, 1H), 7.09 (s, 1H), 6.88 (s, 1H), 6.10 (s, 2H), 5.29 (s, 2H), 4.59 (t, *J* = 6.4 Hz, 2H), 4.05 (s, 3H), 3.28 − 3.22 (m, 2H), 3.16 (t, *J* = 5.5 Hz, 2H), 2.97 − 2.93 (m, 2H), 2.75 (s, 4H), 2.39 − 2.30 (m, 2H), 1.85 (s, 6H), 1.55 − 1.46 (m, 2H), 1.41 − 1.20 (m, 8H), 0.91 − 0.86 (m, 3H). ^13 ^C NMR (75 MHz, CDCl_3_) *δ* 149.73, 149.68, 147.3, 145.2, 144.6, 136.1, 134.4, 133.8, 132.9, 125.4, 121.7, 120.2, 120.1, 109.1, 108.5, 102.2, 71.7, 57.3, 57.1, 53.4, 51.9, 31.7, 31.2, 29.9, 29.5, 29.1, 29.0, 28.6, 26.9, 23.4, 22.5, 14.1. HRMS (ESI) calculated for C_34_H_45_BrN_2_O_4_ [M-Br]^+^ 545.3374, found 545.3383.

10-Methoxy-9–(3-morpholinopropoxy)-13-octyl-5,6-dihydro-[1,3]dioxolo[4,5-g]isoquinolino[3,2-a]isoquinolin-7-ium bromide (**6c**) Yellow solid in 73% yield (94 mg). m.p. 167 − 169 °C. ^1^H NMR (300 MHz, CDCl_3_) *δ* 10.60 (s, 1H), 7.88 (d, *J* = 9.4 Hz, 1H), 7.83 (d, *J* = 9.4 Hz, 1H), 7.08 (s, 1H), 6.87 (s, 1H), 6.09 (s, 2H), 5.29 (s, 2H), 4.57 (t, *J* = 6.6 Hz, 2H), 4.05 (s, 3H), 3.72 (t, *J* = 4.5 Hz, 4H), 3.27 − 3.21 (m, 2H), 3.14 (t, *J* = 5.6 Hz, 2H), 2.67 (t, *J* = 7.4 Hz, 2H), 2.54 (s, 2H), 2.44 (s, 2H), 2.35 − 2.25 (m, 2H), 1.89 − 1.80 (m, 2H), 1.54 − 1.45 (m, 2H), 1.38 − 1.23 (m, 8H), 0.90 − 0.82 (m, 3H). ^13 ^C NMR (75 MHz, CDCl_3_) *δ* 150.2, 149.6, 147.2, 145.4, 145.2, 136.0, 134.3, 133.8, 132.9, 125.3, 122.1, 120.2, 120.1, 109.0, 108.5, 102.1, 73.4, 66.5, 57.5, 57.0, 55.1, 53.4, 31.7, 31.1, 29.9, 29.5, 29.1, 29.0, 28.7, 26.9, 22.5, 14.1. HRMS (ESI) calculated for C_34_H_45_BrN_2_O_5_ [M-Br]^+^ 561.3323, found 561.3334.

9-((6-(Diethylamino)hexyl)oxy)-10-methoxy-13-octyl-5,6-dihydro-[1,3]dioxolo[4,5-g]isoquinolino[3,2-a]isoquinolin-7-ium bromide (**6d**) Yellow solid in 68% yield (91 mg). m.p. 149 − 151 °C. ^1^H NMR (300 MHz, CDCl_3_) *δ* 10.40 (s, 1H), 7.88 (d, *J* = 9.5 Hz, 1H), 7.84 (d, *J* = 9.5 Hz, 1H), 7.08 (s, 1H), 6.87 (s, 1H), 6.09 (s, 2H), 5.26 (s, 2H), 4.48 (t, *J* = 6.7 Hz, 2H), 4.04 (s, 3H), 3.27 − 3.22 (m, 2H), 3.15 (t, *J* = 5.3 Hz, 2H), 2.65 (q, *J* = 7.2 Hz, 4H), 2.59 − 2.54 (m, 2H), 2.09 − 2.00 (m, 2H), 1.89 − 1.79 (m, 2H), 1.64 − 1.52 (m, 4H), 1.49 − 1.38 (m, 4H), 1.34 − 1.23 (m, 8H), 1.08 (t, *J* = 7.2 Hz, 6H), 0.89 − 0.85 (m, 3H). ^13 ^C NMR (75 MHz, CDCl_3_) *δ* 150.2, 149.6, 147.1, 145.0, 144.8, 135.9, 134.4, 133.7, 132.9, 125.4, 122.0, 120.1, 120.0, 108.9, 108.4, 102.0, 74.9, 57.7, 57.0, 51.4, 46.5, 31.6, 31.0, 29.7, 29.4, 29.2, 29.0, 28.8, 28.6, 26.0, 24.7, 22.9, 22.4, 13.9, 8.8. HRMS (ESI) calculated for C_37_H_53_BrN_2_O_4_ [M-Br]^+^ 589.4000, found 589.4021.

10-Methoxy-13-octyl-9-((6-(pyrrolidin-1-yl)hexyl)oxy)-5,6-dihydro-[1,3]dioxolo[4,5-g]isoquinolino[3,2-a]isoquinolin-7-ium bromide (**6e**) Yellow solid in 65% yield (87 mg). m.p. 126 − 128 °C. ^1^H NMR (300 MHz, CDCl_3_) *δ* 10.48 (s, 1H), 7.88 (d, *J* = 9.4 Hz, 1H), 7.83 (d, *J* = 9.4 Hz, 1H), 7.09 (s, 1H), 6.88 (s, 1H), 6.10 (s, 2H), 5.31 (s, 2H), 4.51 (t, *J* = 6.7 Hz, 2H), 4.06 (s, 3H), 3.28 − 3.22 (m, 2H), 3.16 (t, *J* = 5.8 Hz, 2H), 2.72 − 2.63 (m, 6H), 2.11 − 2.02 (m, 2H), 1.85 (s, 6H), 1.73 − 1.65 (m, 2H), 1.62 − 1.57 (m, 2H), 1.54 − 1.43 (m, 4H), 1.36 − 1.24 (m, 8H), 0.91 − 0.86 (m, 3H). ^13 ^C NMR (75 MHz, CDCl_3_) *δ* 150.2, 149.6, 147.1, 144.9, 144.6, 135.9, 134.5, 133.6, 132.9, 125.4, 122.0, 120.1, 120.0, 109.0, 108.4, 102.0, 74.9, 57.7, 57.0, 54.5, 53.2, 31.6, 31.1, 29.8, 29.4, 29.2, 29.0, 28.9, 28.6, 25.8, 24.9, 24.4, 23.2, 22.4, 14.0. HRMS (ESI) calculated for C_37_H_51_BrN_2_O_4_ [M-Br]^+^ 587.3843, found 587.3844.

10-Methoxy-9-((6-morpholinohexyl)oxy)-13-octyl-5,6-dihydro-[1,3]dioxolo[4,5-g]isoquinolino[3,2-a]isoquinolin-7-ium bromide (**6f**) Yellow solid in 77% yield (105 mg). m.p. 164 − 166 °C. ^1^H NMR (300 MHz, CDCl_3_) *δ* 10.52 (s, 1H), 7.87 (d, *J* = 9.4 Hz, 1H), 7.83 (d, *J* = 9.4 Hz, 1H), 7.08 (s, 1H), 6.88 (s, 1H), 6.10 (s, 2H), 5.31 (s, 2H), 4.53 (t, *J* = 6.7 Hz, 2H), 4.05 (s, 3H), 3.71 (t, *J* = 4.6 Hz, 4H), 3.27 − 3.22 (m, 2H), 3.17 (t, *J* = 5.3 Hz, 2H), 2.44 (s, 4H), 2.35 (t, *J* = 7.6 Hz, 2H), 2.13 − 1.98 (m, 4H), 1.90 − 1.81 (m, 2H), 1.63 − 1.39 (m, 6H), 1.34 − 1.24 (m, 8H), 0.90 − 0.86 (m, 3H). ^13 ^C NMR (75 MHz, CDCl_3_) *δ* 150.4, 149.7, 147.2, 145.4, 145.3, 136.0, 134.4, 133.9, 133.0, 125.4, 122.3, 120.3, 120.0, 109.0, 108.5, 102.1, 75.5, 66.4, 58.8, 57.7, 57.0, 53.4, 31.7, 31.2, 29.9 (2), 29.5, 29.1, 29.0, 28.7, 27.1, 25.8, 25.5, 22.5, 14.1. HRMS (ESI) calculated for C_37_H_51_BrN_2_O_5_ [M-Br]^+^ 603.3792, found 603.3802.

10-Methoxy-13-octyl-9-((6-(piperidin-1-yl)hexyl)oxy)-5,6-dihydro-[1,3]dioxolo[4,5-g]isoquinolino[3,2-a]isoquinolin-7-ium bromide (**6g**) Yellow solid in 74% yield (101 mg). m.p. 132 − 134 °C. ^1^H NMR (300 MHz, CDCl_3_) *δ* 10.43 (s, 1H), 7.88 (d, *J* = 9.4 Hz, 1H), 7.84 (d, *J* = 9.4 Hz, 1H), 7.08 (s, 1H), 6.87 (s, 1H), 6.09 (s, 2H), 5.28 (s, 2H), 4.49 (t, *J* = 6.8 Hz, 2H), 4.05 (s, 3H), 3.27 − 3.22 (m, 2H), 3.16 (t, *J* = 5.5 Hz, 2H), 2.55 − 2.45 (m, 6H), 2.09 − 2.00 (m, 2H), 1.89 − 1.80 (m, 2H), 1.67 − 1.59 (m, 6H), 1.57 − 1.38 (m, 8H), 1.32 − 1.23 (m, 8H), 0.89 − 0.85 (m, 3H). ^13 ^C NMR (75 MHz, CDCl_3_) *δ* 150.2, 149.5, 147.0, 145.0, 144.8, 135.7, 134.3, 133.5, 132.8, 125.4, 122.0, 120.1, 120.0, 108.9, 108.3, 102.0, 75.1, 58.7, 57.5, 56.9, 53.9, 31.5, 31.0, 29.7 (2), 29.3, 29.0, 28.8, 28.5, 27.0, 25.7, 25.2, 25.0, 23.6, 22.4, 13.9. HRMS (ESI) calculated for C_38_H_53_BrN_2_O_4_ [M-Br]^+^ 601.4000, found 601.4011.

10-Methoxy-9-((6–(4-methylpiperazin-1-yl)hexyl)oxy)-13-octyl-5,6-dihydro-[1,3]dioxolo[4,5-g]isoquinolino[3,2-a]isoquinolin-7-ium bromide (**6h**) Yellow solid in 63% yield (88 mg). m.p. 165 − 167 °C. ^1^H NMR (300 MHz, CDCl_3_) *δ* 10.50 (s, 1H), 7.87 (d, *J* = 9.5 Hz, 1H), 7.82 (d, *J* = 9.5 Hz, 1H), 7.08 (s, 1H), 6.88 (s, 1H), 6.10 (s, 2H), 5.29 (s, 2H), 4.52 (t, *J* = 6.7 Hz, 2H), 4.05 (s, 3H), 3.27 − 3.22 (m, 2H), 3.17 (t, *J* = 5.9 Hz, 2H), 2.48 (s, 8H), 2.40 − 2.35 (m, 2H), 2.28 (s, 3H), 2.11 − 2.02 (m, 2H), 1.90 − 1.80 (m, 2H), 1.60 − 1.48 (m, 4H), 1.46 − 1.24 (m, 12H), 0.90 − 0.86 (m, 3H). ^13 ^C NMR (75 MHz, CDCl_3_) *δ* 150.3, 149.6, 147.1, 145.2, 145.0, 135.9, 134.4, 133.8, 132.9, 125.4, 122.2, 120.2, 120.1, 109.0, 108.5, 102.1, 75.3, 58.5, 57.7, 57.0, 54.7, 52.9, 45.8, 31.6, 31.1, 29.9 (2), 29.5, 29.1, 28.9, 28.6, 27.2, 26.5, 25.5, 22.5, 14.0. HRMS (ESI) calculated for C_38_H_54_BrN_3_O_4_ [M-Br]^+^ 616.4109, found 616.4114.

10-Methoxy-9-((8-morpholinooctyl)oxy)-13-octyl-5,6-dihydro-[1,3]dioxolo[4,5-g]isoquinolino[3,2-a]isoquinolin-7-ium bromide (**6i**) Yellow solid in 72% yield (102 mg). m.p. 147 − 149 °C. ^1^H NMR (300 MHz, CDCl_3_) *δ* 10.42 (s, 1H), 7.88 (d, *J* = 9.4 Hz, 1H), 7.83 (d, *J* = 9.4 Hz, 1H), 7.08 (s, 1H), 6.89 (s, 1H), 6.10 (s, 2H), 5.27 (s, 2H), 4.49 (t, *J* = 6.7 Hz, 2H), 4.05 (s, 3H), 3.84 (s, 4H), 3.28 − 3.22 (m, 2H), 3.18 (t, *J* = 5.3 Hz, 2H), 2.72 (s, 4H), 2.63 − 2.57 (m, 2H), 2.09 − 2.00 (m, 2H), 1.91 − 1.80 (m, 2H), 1.68 − 1.59 (m, 2H), 1.55 − 1.49 (m, 4H), 1.37 − 1.24 (m, 14H), 0.90 − 0.86 (m, 3H). ^13 ^C NMR (75 MHz, CDCl_3_) *δ* 150.4, 149.6, 147.2, 145.4, 145.1, 136.0, 134.4, 133.9, 133.0, 125.4, 122.2, 120.2, 120.0, 109.0, 108.6, 102.1, 75.5, 65.8, 58.6, 57.8, 57.0, 53.0, 31.7, 31.2, 30.0, 29.9, 29.5, 29.13, 29.05, 29.0, 28.7, 27.0, 25.5, 25.3, 22.6, 14.1. HRMS (ESI) calculated for C_39_H_55_BrN_2_O_5_ [M-Br]^+^ 631.4105, found 631.4117.

10-Methoxy-13-octyl-9-((8-(piperidin-1-yl)octyl)oxy)-5,6-dihydro-[1,3]dioxolo[4,5-g]isoquinolino[3,2-a]isoquinolin-7-ium bromide (**6j**) Yellow solid in 75% yield (106 mg). m.p. 144 − 146 °C. ^1^H NMR (300 MHz, CDCl_3_) *δ* 10.47 (s, 1H), 7.88 (d, *J* = 9.4 Hz, 1H), 7.83 (d, *J* = 9.4 Hz, 1H), 7.09 (s, 1H), 6.89 (s, 1H), 6.10 (s, 2H), 5.30 (s, 2H), 4.52 (t, *J* = 6.8 Hz, 2H), 4.06 (s, 3H), 3.28 − 3.22 (m, 2H), 3.18 (t, *J* = 5.6 Hz, 2H), 2.51 (s, 4H), 2.44 − 2.39 (m, 2H), 2.10 − 2.00 (m, 2H), 1.91 − 1.81 (m, 2H), 1.70 − 1.62 (m, 4H), 1.59 − 1.45 (m, 6H), 1.39 − 1.24 (m, 16H), 0.91 − 0.87 (m, 3H). ^13 ^C NMR (75 MHz, CDCl_3_) *δ* 150.3, 149.6, 147.1, 145.2, 144.9, 135.9, 134.4, 133.7, 132.9, 125.5, 122.1, 120.2, 120.0, 109.0, 108.5, 102.1, 75.4, 58.9, 57.8, 57.0, 54.1, 31.7, 31.1, 29.9, 29.8, 29.5, 29.2, 29.1, 28.9, 28.6, 27.3, 25.9, 25.5, 25.0, 23.7, 22.5, 14.0. HRMS (ESI) calculated for C_40_H_57_BrN_2_O_4_ [M-Br]^+^ 629.4313, found 629.4323.

10-Methoxy-9-((8–(4-methylpiperazin-1-yl)octyl)oxy)-13-octyl-5,6-dihydro-[1,3]dioxolo[4,5-g]isoquinolino[3,2-a]isoquinolin-7-ium bromide (**6k**) Yellow solid in 66% yield (96 mg). m.p. 151 − 153 °C. ^1^H NMR (300 MHz, CDCl_3_) *δ* 10.50 (s, 1H), 7.87 (d, *J* = 9.3 Hz, 1H), 7.83 (d, *J* = 9.3 Hz, 1H), 7.09 (s, 1H), 6.89 (s, 1H), 6.10 (s, 2H), 5.30 (s, 2H), 4.53 (t, *J* = 6.7 Hz, 2H), 4.05 (s, 3H), 3.28 − 3.22 (m, 2H), 3.18 (t, *J* = 5.4 Hz, 2H), 2.48 (s, 8H), 2.34 (t, *J* = 7.8 Hz, 2H), 2.29 (s, 3H), 2.11 − 2.01 (m, 2H), 1.91 − 1.81 (m, 2H), 1.56 − 1.47 (m, 6H), 1.39 − 1.24 (m, 14H), 0.91 − 0.87 (m, 3H). ^13 ^C NMR (75 MHz, CDCl_3_) *δ* 150.3, 149.6, 147.1, 145.2, 144.8, 135.9, 134.4, 133.7, 132.9, 125.4, 122.1, 120.1, 120.0, 108.9, 108.5, 102.0, 75.3, 57.7, 57.3, 57.0, 52.6, 51.0, 44.6, 31.6, 31.1, 29.8 (2), 29.4, 29.0, 28.90, 28.85, 28.8, 28.6, 26.7, 25.4, 24.9, 22.5, 14.0. HRMS (ESI) calculated for C_40_H_58_BrN_3_O_4_ [M-Br]^+^ 644.4422, found 644.4429.

10-Methoxy-9-((10-morpholinodecyl)oxy)-13-octyl-5,6-dihydro-[1,3]dioxolo[4,5-g]isoquinolino[3,2-a]isoquinolin-7-ium bromide (**6l**) Yellow solid in 70% yield (104 mg). m.p. 135 − 137 °C. ^1^H NMR (300 MHz, CDCl_3_) *δ* 10.44 (s, 1H), 7.88 (d, *J* = 9.4 Hz, 1H), 7.83 (d, *J* = 9.4 Hz, 1H), 7.09 (s, 1H), 6.89 (s, 1H), 6.10 (s, 2H), 5.28 (s, 2H), 4.51 (t, *J* = 6.8 Hz, 2H), 4.05 (s, 3H), 3.82 (s, 4H), 3.28 − 3.22 (m, 2H), 3.19 (t, *J* = 5.5 Hz, 2H), 2.65 (s, 4H), 2.54 − 2.49 (m, 2H), 2.09 − 1.99 (m, 2H), 1.91 − 1.80 (m, 2H), 1.61 − 1.51 (m, 6H), 1.41 − 1.24 (m, 18H), 0.90 − 0.86 (m, 3H). ^13 ^C NMR (75 MHz, CDCl_3_) *δ* 150.2, 149.5, 147.1, 145.0, 144.7, 135.8, 134.4, 133.6, 132.8, 125.4, 122.0, 120.1, 120.0, 108.9, 108.4, 102.0, 75.3, 65.9, 58.6, 57.7, 56.9, 53.0, 31.6, 31.0, 29.9, 29.7, 29.4, 29.3, 29.21, 29.15, 29.0, 28.8, 28.5, 27.0, 25.52, 25.48, 22.4, 13.9. HRMS (ESI) calculated for C_41_H_59_BrN_2_O_5_ [M-Br]^+^ 659.4418, found 659.4428.

10-Methoxy-13-octyl-9-((10-(piperidin-1-yl)decyl)oxy)-5,6-dihydro-[1,3]dioxolo[4,5-g]isoquinolino[3,2-a]isoquinolin-7-ium bromide (**6m**) Yellow solid in 67% yield (99 mg). m.p. 142 − 144 °C. ^1^H NMR (300 MHz, CDCl_3_) *δ* 10.4 (s, 1H), 7.88 (d, *J* = 9.5 Hz, 1H), 7.83 (d, *J* = 9.5 Hz, 1H), 7.08 (s, 1H), 6.89 (s, 1H), 6.10 (s, 2H), 5.28 (s, 2H), 4.51 (t, *J* = 6.7 Hz, 2H), 4.05 (s, 3H), 3.27 − 3.22 (m, 2H), 3.18 (t, *J* = 5.8 Hz, 2H), 2.42 (m, 4H), 2.35 − 2.30 (m, 2H), 2.09 − 1.99 (m, 2H), 1.90 − 1.80 (m, 2H), 1.65 − 1.58 (m, 4H), 1.55 − 1.46 (m, 6H), 1.41 − 1.28 (m, 20H), 0.90 − 0.86 (m, 3H). ^13 ^C NMR (75 MHz, CDCl_3_) *δ* 150.3, 149.6, 147.1, 145.1, 144.8, 135.9, 134.5, 133.7, 132.9, 125.5, 122.1, 120.15, 120.06, 108.9, 108.5, 102.1, 75.4, 59.0, 57.8, 57.0, 54.0, 31.6, 31.1, 30.0, 29.8, 29.42, 29.36, 29.3, 29.0, 28.9, 28.6, 27.4, 25.9, 25.6, 25.0, 23.7, 22.5, 14.0. HRMS (ESI) calculated for C_42_H_61_BrN_2_O_4_ [M-Br]^+^ 657.4626, found 657.4636.

10-Methoxy-9-((10–(4-methylpiperazin-1-yl)decyl)oxy)-13-octyl-5,6-dihydro-[1,3]dioxolo[4,5-g]isoquinolino[3,2-a]isoquinolin-7-ium bromide (**6n**) Yellow solid in 62% yield (93 mg). m.p. 136 − 138 °C. ^1^H NMR (300 MHz, CDCl_3_) *δ* 10.49 (s, 1H), 7.88 (d, *J* = 9.6 Hz, 1H), 7.83 (d, *J* = 9.3 Hz, 1H), 7.09 (s, 1H), 6.89 (s, 1H), 6.10 (s, 2H), 5.30 (s, 2H), 4.53 (t, *J* = 6.7 Hz, 2H), 4.06 (s, 3H), 3.28 − 3.22 (m, 2H), 3.19 (t, *J* = 6.0 Hz, 2H), 2.48 (s, 8H), 2.33 (t, *J* = 7.8 Hz, 2H), 2.29 (s, 3H), 2.10 − 2.01 (m, 2H), 1.92 − 1.81 (s, 2H), 1.56 − 1.46 (m, 6H), 1.38 − 1.25 (m, 18H), 0.91 − 0.87 (m, 3H). ^13 ^C NMR (75 MHz, CDCl_3_) *δ* 150.3, 149.6, 147.1, 145.2, 145.0, 135.9, 134.4, 133.7, 132.9, 125.4, 122.1, 120.2, 120.0, 108.9, 108.5, 102.0, 75.4, 58.6, 57.7, 57.0, 54.8, 52.9, 45.8, 31.6, 31.1, 30.0, 29.8, 29.4, 29.2, 29.1, 28.9, 28.6, 27.4, 26.6, 25.6, 22.5, 14.0. HRMS (ESI) calculated for C_42_H_62_BrN_3_O_4_ [M-Br]^+^ 672.4735, found 672.4743.

### 4.2. Pharmacology

#### 4.2.1. *In vitro* antiproliferative assay

HepG2, Sk-Hep-1, Huh-7, Hep3B and L-02 cells were purchased from Nanjing Key Gen Biotech Co. Ltd. (Nanjing, China). The cytotoxicity of test compounds was determined using an MTT assay. Briefly, the cell lines were incubated at 37 °C in a humidified 5% CO_2_ incubator for 24 h in 96-microwell plates. After medium removal, 100 μL of culture medium with 0.1% DMSO containing test compounds at different concentrations was added to each well and incubated at 37 °C for another 72 h. The MTT (5 mg/mL in PBS) was added and incubated for another 4 h, the optical density was detected with a microplate reader at 490 nm. The IC_50_ values were calculated according to the dose-dependent curves. All the experiments were repeated in at least three independent experiments.

#### 4.2.2. Cell cycle analysis

HepG2 cells were seeded into 6-well plates and incubated at 37 °C in a humidified 5% CO_2_ incubator for 24 h, and then treated with or without **6k** at indicated concentrations for another 72 h. The collected cells were fixed by adding 70% ethanol at 4 °C for 12 h. Subsequently, the cells were resuspended in PBS containing 100 μL RNase A and 400 μL of PI for 30 min. The DNA content of the cells was measured using a FACS Calibur flow cytometer (BectoneDickinson, San Jose, CA, USA).

#### 4.2.3. Fret-melting assay

The fluorescent labelled FPu18T (5′-FAM-AGGGTGGGGA-GGGTGGGG-TAMRA-3′) and F10T [5′-FAM-dTATAGCTATA-HEG-TATA-GCTATA-TAMRA-3′] (HEG linker: [(-CH_2_-CH_2_-O-)_6_]) were employed as *c-*MYC G4 and duplex DNA model respectively. The experiments were performed in a Real-Time PCR apparatus (Roche LigntCycler). The concentrations of test compounds were diluted by Tris-HCl buffer (0.2 mM KCl contained, *p*H 7.2) to 0.2 μM. The melting process of G4 DNA was monitored in the presence or absence of 2 μM compounds by measuring the fluorescence intensity of fluorescein. Measurements were performed with excitation at 470 nm and detection at 530 nm. Fluorescence readings were taken at an interval of 2 °C over the range of 30–98 °C, with a constant temperature being maintained for 1 min prior to each reading to ensure a stable value. The data was analysed by Origin 8.5 (OriginLab Corp.). To test the selectivity of compounds for G4 structure, 10 μM ds26 DNA (5′-GTTAGCCTAGCTTAAGCTAGGCTAAC-3′) was added, followed by the same method as above.

#### 4.2.4. Cell apoptosis analysis

Briefly, after treatment with or without **6k** at indicated concentrations for 72 h, the cells were washed twice in PBS, centrifuged and resuspended in 500 μL AnnexinV binding buffer. The cells were then harvested, washed and stained with 5 μL Annexin V-FITC and 5 μL PI in the darkness for 15 min. Apoptosis was analysed using a FACS Calibur flow cytometer (BectoneDickinson, San Jose, CA, USA).

#### 4.2.5. MMP measurement

HepG2 cells were incubated with **6k** as described above. After incubation for 72 h, the cells were washed in PBS and resuspended in 500 μL JC-1 incubation buffer at 37 °C for 15 min. Then, **6k** was immediately assessed for a red fluorescence using a microplate reader (ELx80, Bio-Tek, USA). The fluorescent signal of monomers was measured with an excitation wavelength of 488 nm. The percentage of cells with healthy or collapsed mitochondrial membrane potentials was monitored by flow cytometry analysis (BectoneDickinson, San Jose, CA, USA).

#### 4.2.6. Western blotting analysis

HepG2 cells were incubated with different doses of **6k** for 72 h. Cells were collected, centrifuged, and washed two times with ice-cold PBS. The pellet was then re-suspended in lysis buffer. After the cells were lysed on ice for 20 min, lysates were centrifuged at 13000 *g* at 4 °C for 15 min. The protein concentration in the supernatant was determined using the BCA protein assay reagents. Equal amounts of protein (20 *μ*g) were resolved using sodium dodecyl sulfate-polyacrylamide gel electrophoresis (SDS-PAGE) (10–12% acrylamide gels) and transferred to PVDF membrane. Membranes were blocked for 1 h at room temperature. Membranes were then incubated with primary antibodies against *p*-cdc25c, cdc2, cyclin B1, Bax, Bcl-2, cytochrome C, caspase-3, VDAC1 and *β*-actin, the membrane being gently rotated overnight at 4 °C. The bound antibodies were detected using horseradish peroxidase (HRP)-conjugated second antibodies and visualised by the enhanced chemiluminescent reagent.

#### 4.2.7. Determination of the LWP coefficient and aqueous solubility

##### 4.2.7.1. LWP coefficient (log D) measurement

Berberine, **3c** and **6k** of 1 mg were dissolved in three centrifugal tubes (5 ml) containing phosphate buffer (pH 7.4)-saturated 1-octanol solution (2 mL), followed by the addition of 1-octanol saturated PBS (2 mL). Each tube was vigorously mixed for 1 min and then shaken for 6 h at a speed of 1000 rpm at 37 °C. The mixtures were then centrifuged. 1 mL of 1-octanol phase and 1 mL of buffer were separately collected. Each phase was diluted to a suitable concentration and analysed by HPLC (Shimadzu DGU-20A3R) on a Shimadzu-GL WondaSil C18-WR column (*λ* = 254 nm) with an eluent of methanol/water (65/35, v/v, 1.0 ml/min). The log D value for each compound was calculated from the ratio of the peak areas using the following equation: Log D = log (Area_oct_ × x/Area_buf_ × y). x, y is the dilution factor.

##### 4.2.7.2. Aqueous solubility measurement

Berberine, **3c** and **6k** (5 mg) were dissolved in 1 ml PBS (50 mM, pH 7.4) at 25 °C. The suspension was sonicated for 30 min and then centrifuged at 15000 g for 5 min. The supernatant was filtered through a PTFE filter (0.2 μm) slowly. Then 10 μL filtered solution was injected into the HPLC system (Shimadzu DGU-20A3R) on a Shimadzu-GL WondaSil C18-WR column and quantified using the standard curves made by the corresponding known concentrations of berberine, **3c** and **6k**. The eluent was methanol/water (65/35, v/v) at a flow rate of 1.0 ml/min with the detection wavelength at 254 nm.

#### 4.2.8. *In vivo* anti-HCC assay

Five-week-old male Institute of Cancer Research (ICR) mice were purchased from Shanghai SLAC Laboratory Animals Co. Ltd. Briefly, a total of 1 × 10^6^ H22 cells were subcutaneously inoculated into the right flank of ICR mice according to protocols of tumour transplant research, to initiate tumour growth. After incubation for one day, mice were weighed and randomly divided into 5 groups of 8 animals. The groups were administered berberine (30 mg/kg) and **6k** (15 or 30 mg/kg) in a vehicle of 10% DMF/2% Tween 80/88% saline, respectively. Vehicle and cyclophosphamide (20 mg/kg) were employed as negative and positive controls, respectively. Treatments were performed by intravenous injection once per day for a total of 21 consecutive days. Body weights and tumour volumes were measured every 2 days. The mice were sacrificed after the treatments, and the tumours were excised and weighed. The tumour volume and inhibition rate (*w*/*w*) were calculated as follows: tumour volume = *L* × *W*^2^/2, where *L* is the length and *W* is the width; tumour inhibitory ratio (%) = (1-average tumour weight of treated group/average tumour weight of control group) × 100%. All procedures were performed following institutional approval in accordance with the NIH Guide for the Care and Use of Laboratory Animals.

## Supplementary Material

Supplemental Material
