# Supplementary material for: Development of novel 9-O-substituted-13-octylberberine derivatives as potential anti-hepatocellular carcinoma agents
Source: J Enzyme Inhib Med Chem. 2022 Sep 6;37(1):2423–33. doi: 10.1080/14756366.2022.2118268 (PMC9467586; doi:10.1080/14756366.2022.2118268)
Supplement: Supplemental Material [file IENZ_A_2118268_SM8008.pdf]

Supporting Information

Development of novel 9-*O*-substituted-13-octylberberine derivatives as potential anti-hepatocellular carcinoma agents

Jichao Chen [a,*], Yiping Duan [b], Xiaoxuan Yu [c], Jiarou Zhong [a], Jing Bai [d], Nian-Guang Li [a,*], Zheying Zhu [e] , Jinyi Xu [b,*]

[a] School of Pharmacy, Nanjing University of Chinese Medicine, Nanjing 210023, P. R. China

[b] State Key Laboratory of Natural Medicines and Department of Medicinal Chemistry, China Pharmaceutical University, 24 Tong Jia Xiang, Nanjing 210009, P. R. China

[c] School of Medicine•Holistic Integrative Medicine, Nanjing University of Chinese Medicine, Nanjing 210023, P. R. China

[d] School of Chemistry and Life Sciences, Suzhou University of Science and Technology, Suzhou 215009, P. R. China

[e] Division of Molecular Therapeutics & Formulation, School of Pharmacy, the University of Nottingham, University Park Campus, Nottingham NG7 2RD, U.K.

[*]**Corresponding authors:** E-Mail: chenjichao@njucm.edu.cn (J. Chen);
E-Mail: linianguang@njucm.edu.cn (N. Li);
E-Mail: jinyixu@china.com (J. Xu).

Copies of ^{1}H and ^{13}C NMR spectra for the title compounds

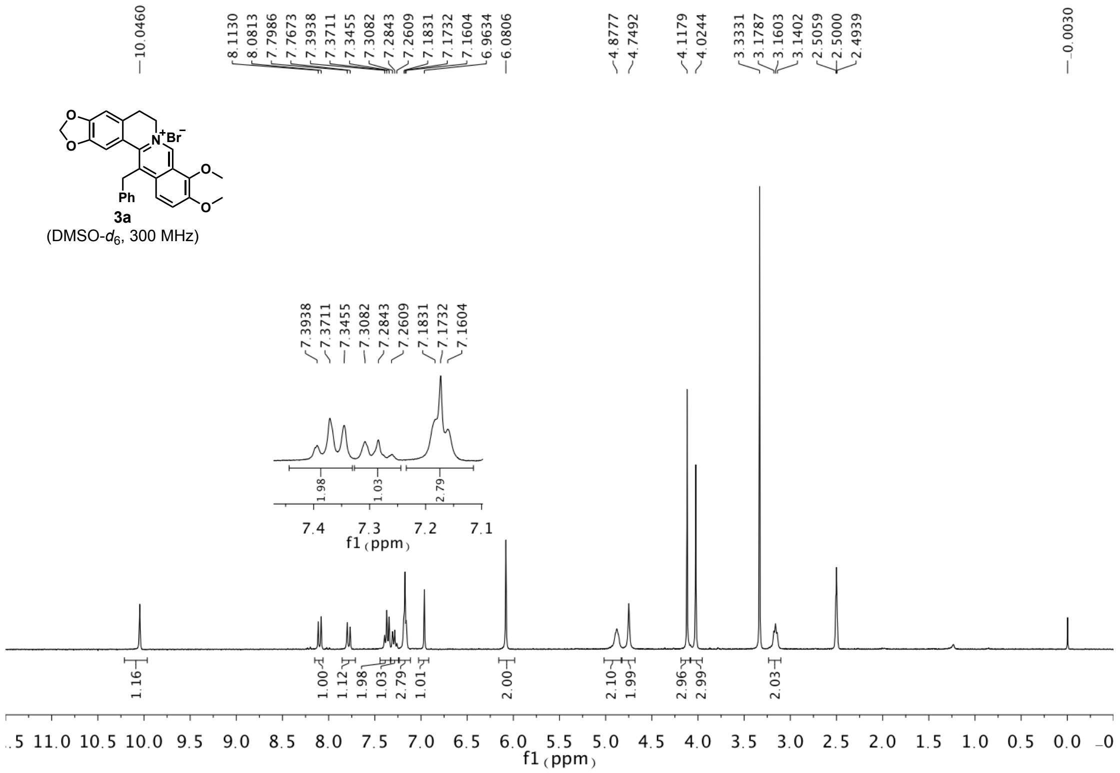

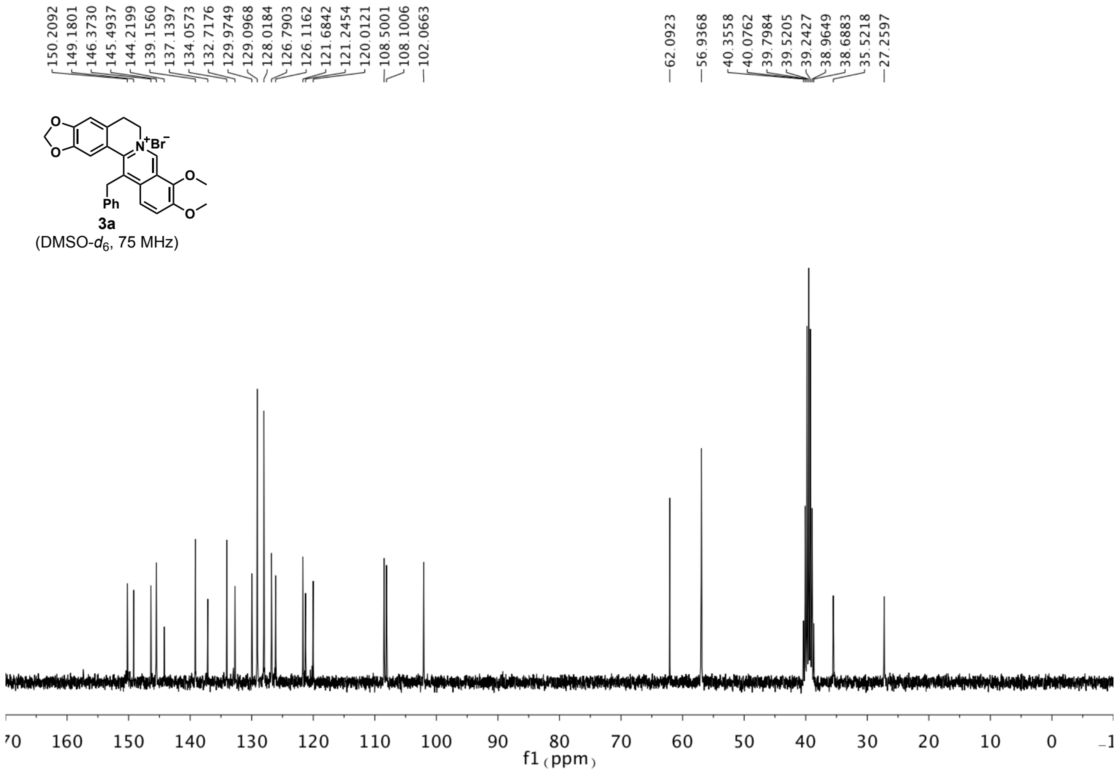

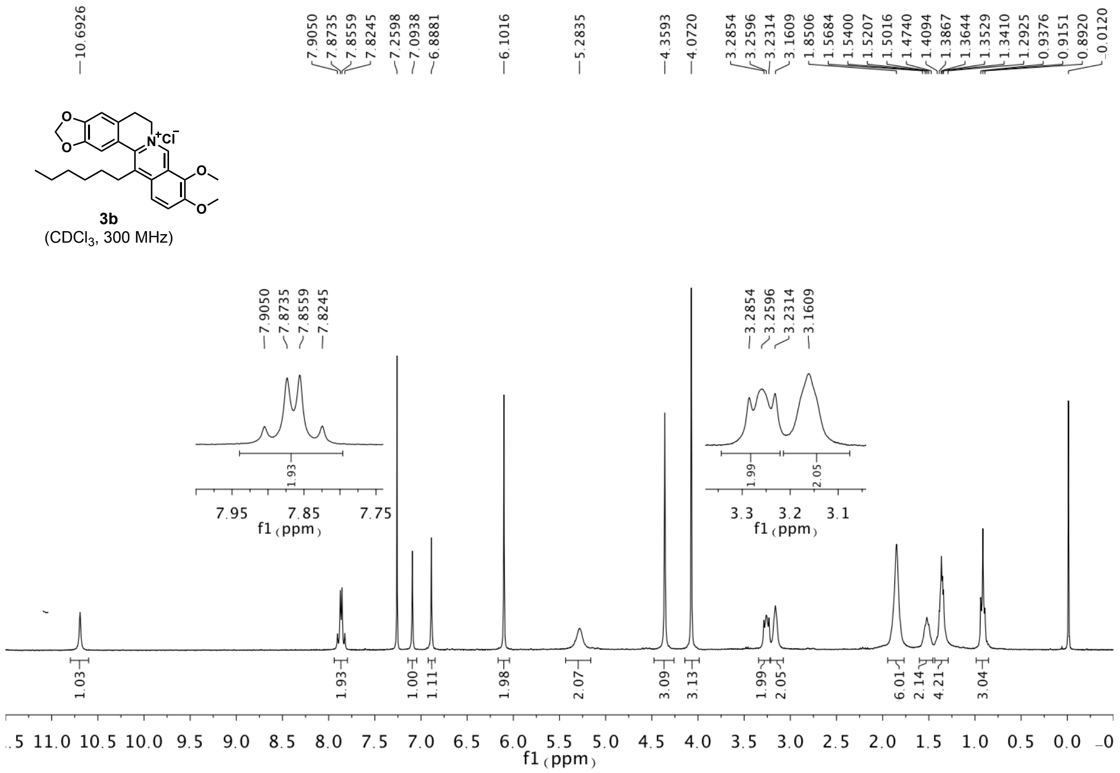
3b
(CDCl$_3$, 300 MHz)
f1 (ppm)

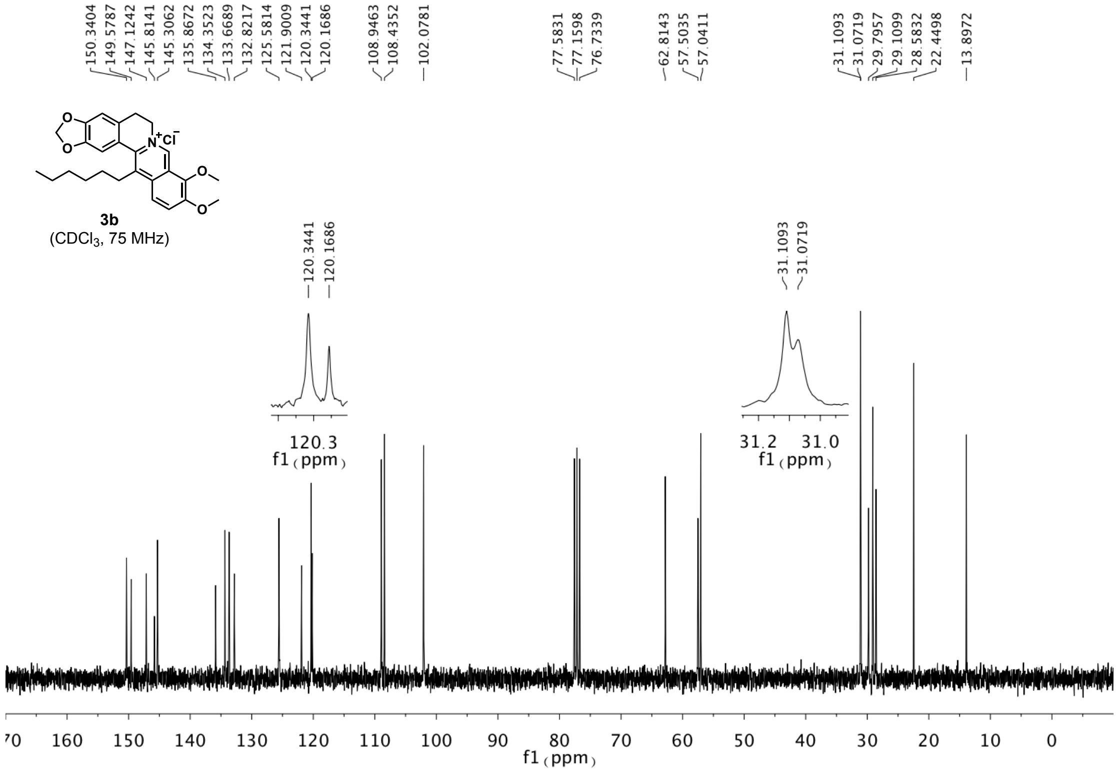
3b
(CDCl$_3$, 75 MHz)
f1 (ppm)

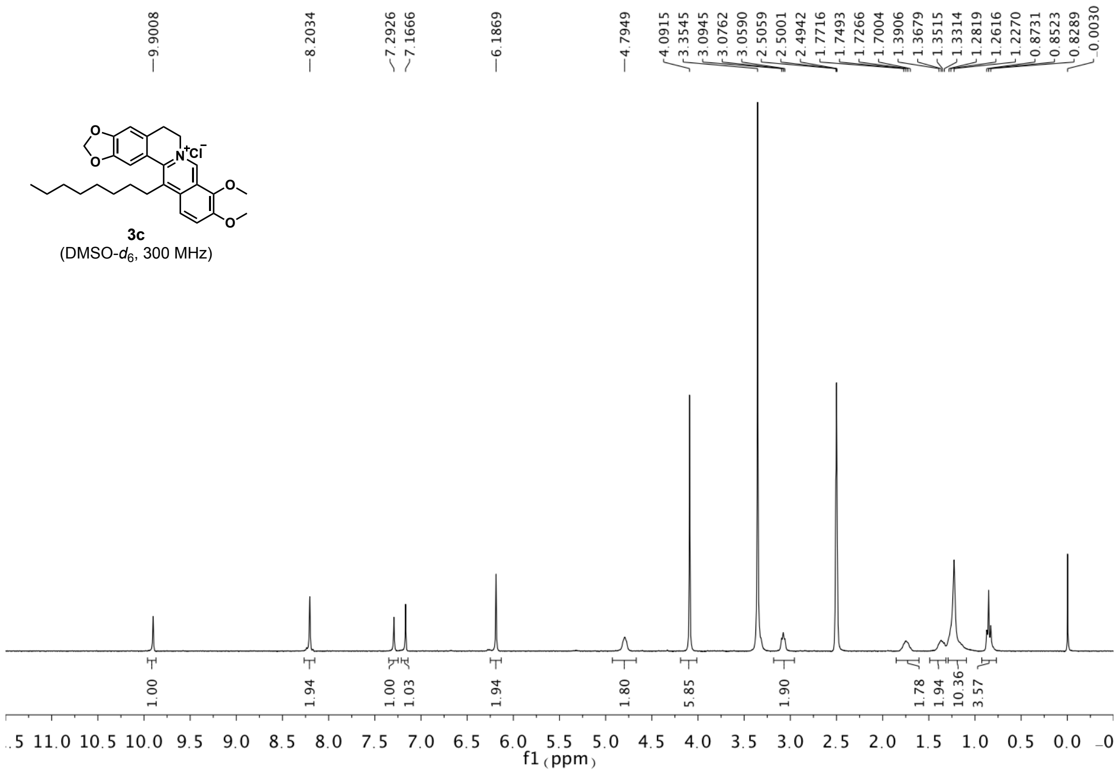
3c
(DMSO-d_6, 300 MHz)
9.9008
8.2034
7.2926
7.1666
6.1869
4.7949
4.0915
3.3545
3.0945
3.0762
3.0590
2.5059
2.5001
2.4942
1.7716
1.7493
1.7266
1.7004
1.3906
1.3679
1.3515
1.3314
1.2819
1.2616
1.2270
0.8731
0.8523
0.8289
0.0030
1.00
1.94
1.00
1.03
1.94
1.80
5.85
1.90
1.78
1.94
10.36
3.57
f1 (ppm)

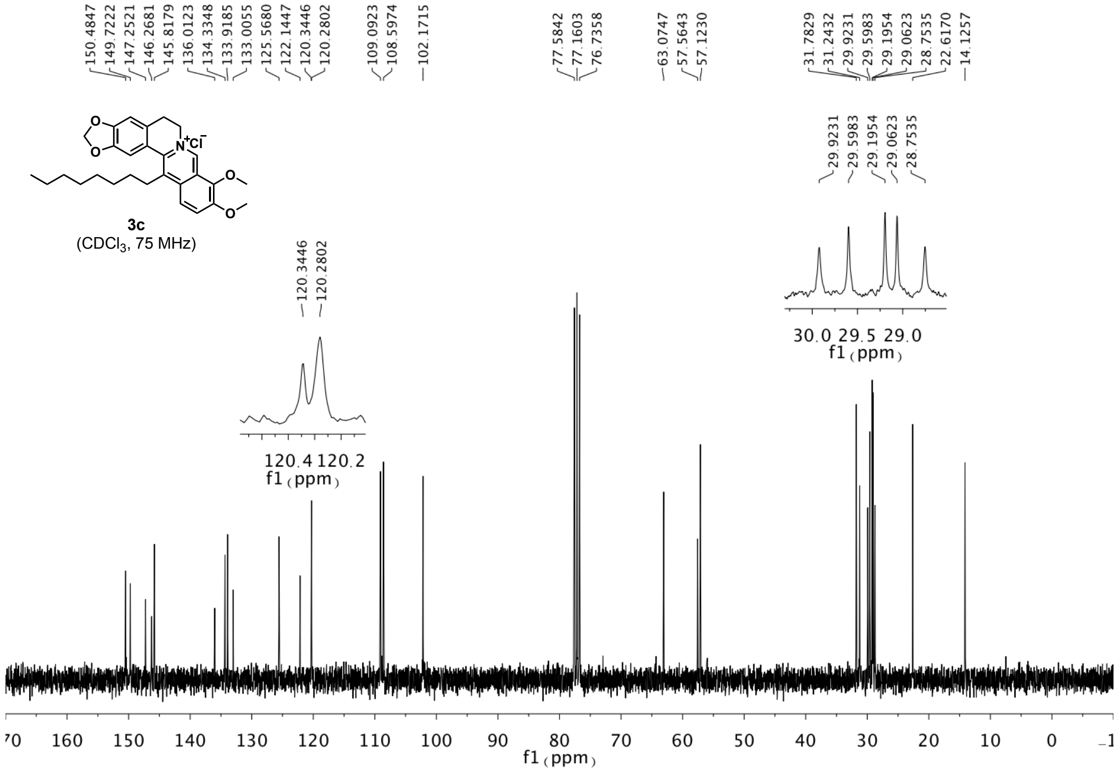
3c
(CDCl$_3$, 75 MHz)
150.4847
149.7222
147.2521
146.2681
145.8179
136.0123
134.3348
133.9185
133.0055
125.5680
122.1447
120.3446
120.2802
109.0923
108.5974
102.1715
77.5842
77.1603
76.7358
63.0747
57.5643
57.1230
31.7829
31.2432
29.9231
29.5983
29.1954
29.0623
28.7535
22.6170
14.1257
f1 (ppm)

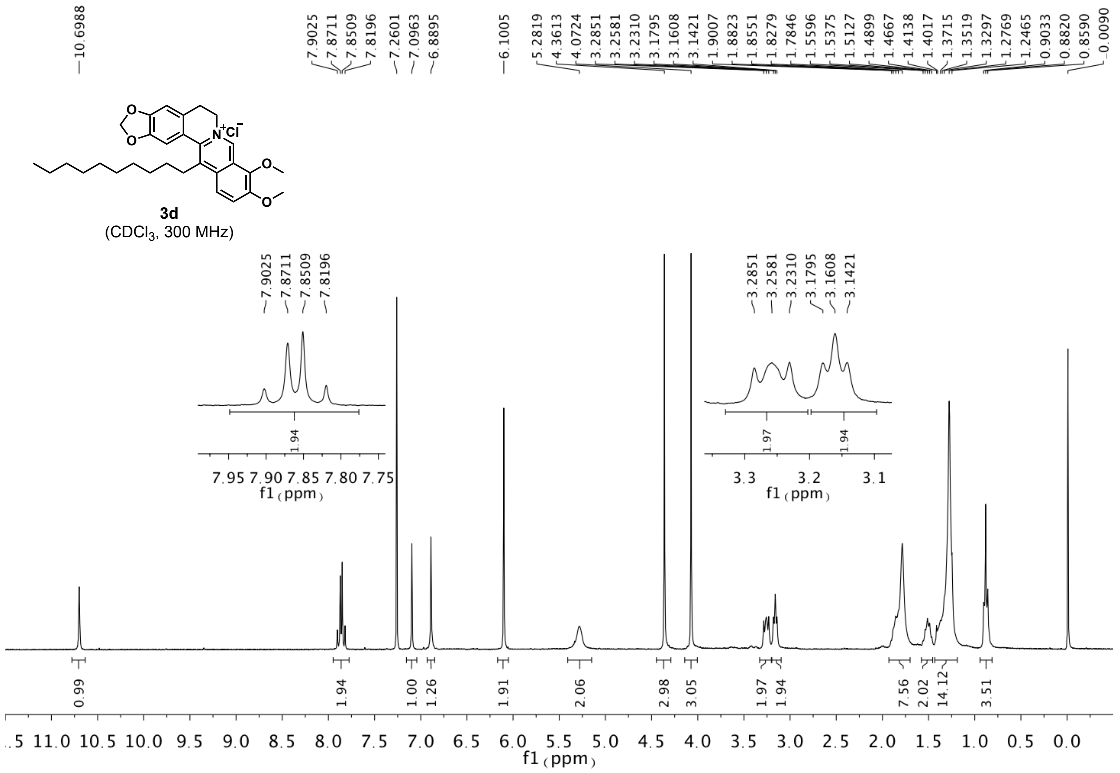
3d
(CDCl3, 300 MHz)
f1 (ppm)

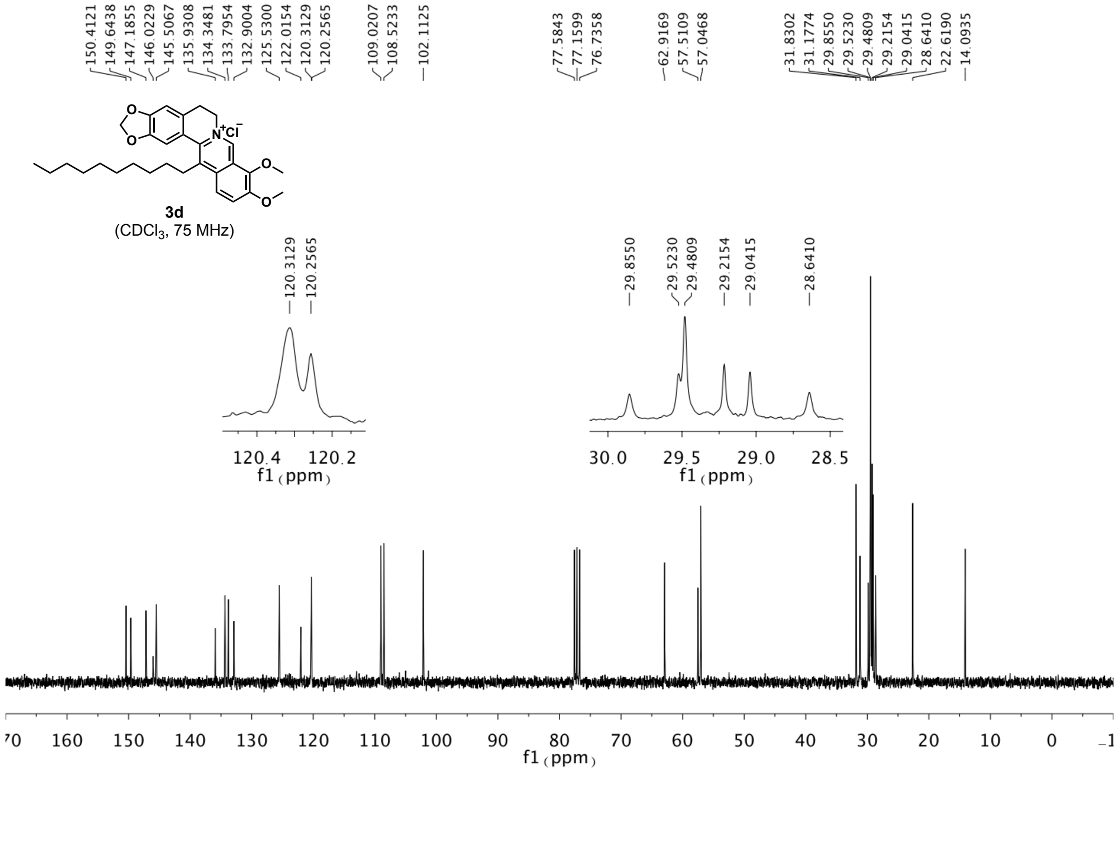
3d
(CDCl3, 75 MHz)
f1 (ppm)

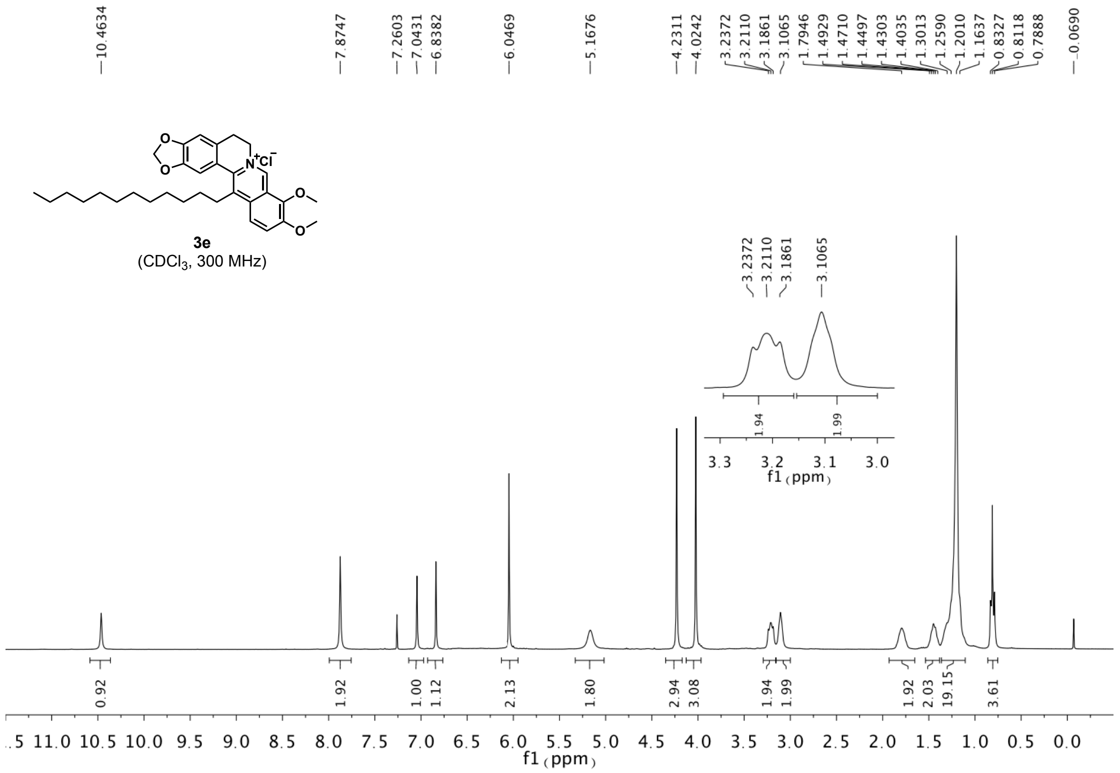
3e
(CDCl$_3$, 300 MHz)
10.4634
7.8747
7.2603
7.0431
6.8382
6.0469
5.1676
4.2311
4.0242
3.2372
3.2110
3.1861
3.1065
1.7946
1.4929
1.4710
1.4497
1.4303
1.4035
1.3013
1.2590
1.2010
1.1637
0.8327
0.8118
0.7888
-0.0690
0.92
1.92
1.00
1.12
2.13
1.80
2.94
3.08
1.94
1.99
1.92
2.03
19.15
3.61
f1 (ppm)

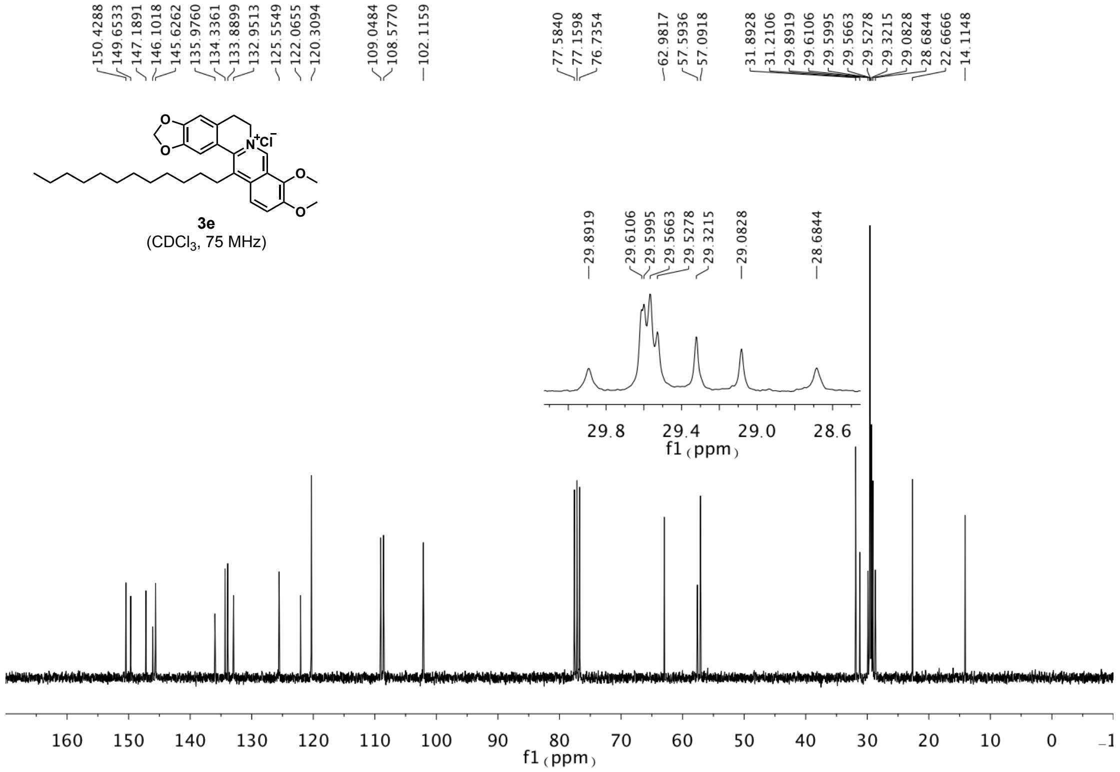
3e
(CDCl$_3$, 75 MHz)
150.4288
149.6533
147.1891
146.1018
145.6262
135.9760
134.3361
133.8899
132.9513
125.5549
122.0655
120.3094
109.0484
108.5770
102.1159
77.5840
77.1598
76.7354
62.9817
57.5936
57.0918
31.8928
31.2106
29.8919
29.6106
29.5995
29.5663
29.5278
29.3215
29.0828
28.6844
22.6666
14.1148
f1 (ppm)

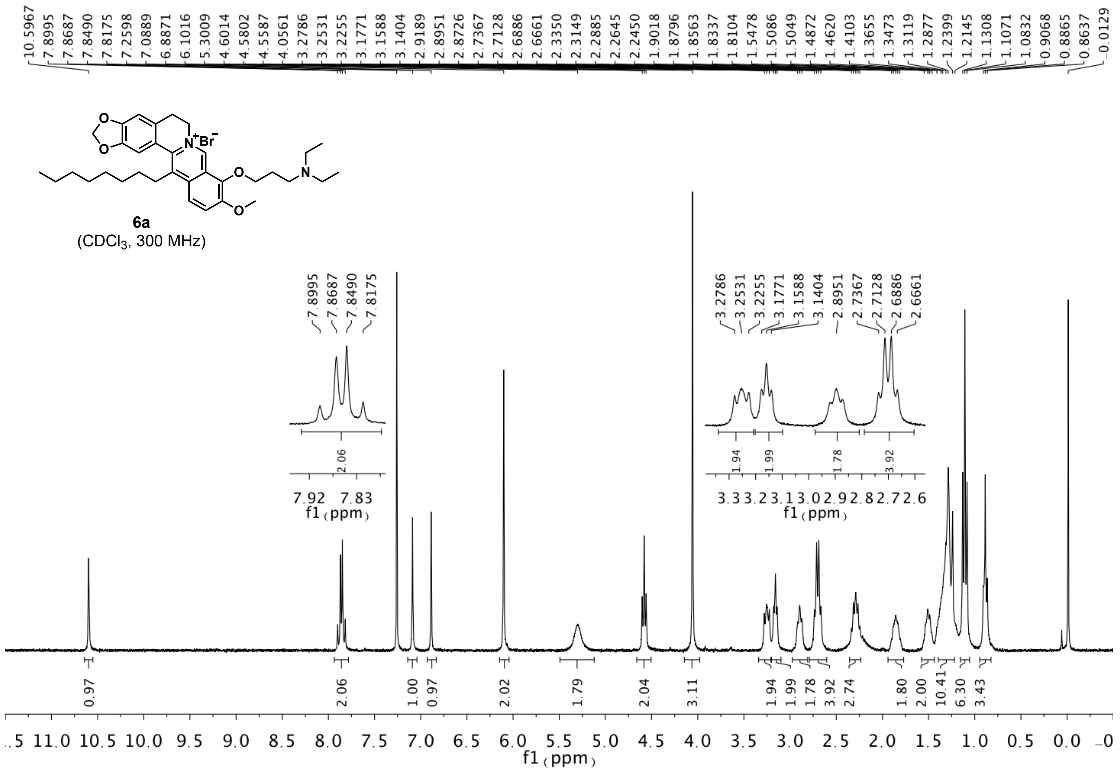
10.5967 7.8995 7.8687 7.8490 7.8175 7.2598 7.0889 6.8871 6.1016 5.3009 4.6014 4.5802 4.5587 4.0561 3.2786 3.2531 3.2255 3.1771 3.1588 3.1404 2.9189 2.8951 2.8726 2.7367 2.7128 2.6886 2.6661 2.3350 2.3149 2.2885 2.2645 2.2450 1.9018 1.8796 1.8563 1.8337 1.8104 1.5478 1.5086 1.5049 1.4872 1.4620 1.4103 1.3655 1.3473 1.3119 1.2877 1.2399 1.2145 1.1308 1.1071 1.0832 0.9068 0.8865 0.8637 0.0129
6a
(CDCl$_3$, 300 MHz)
f1 (ppm)

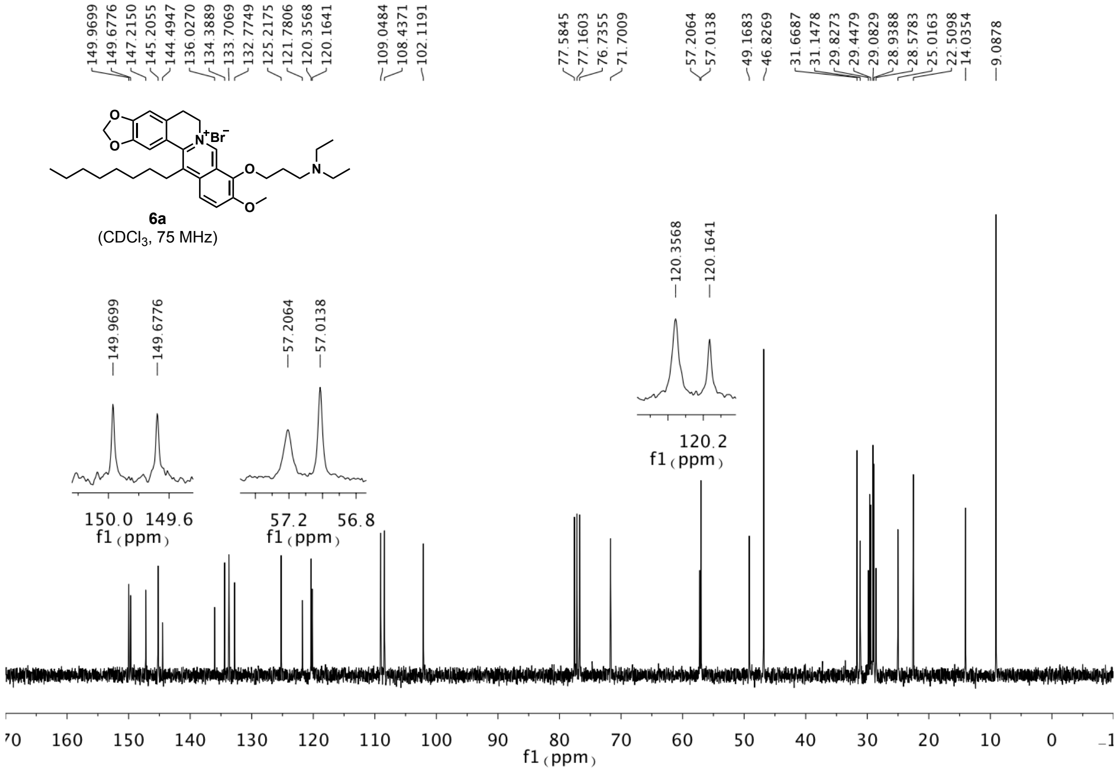
149.9699 149.6776 147.2150 145.2055 144.4947 136.0270 134.3889 133.7069 132.7749 125.2175 121.7806 120.3568 120.1641 109.0484 108.4371 102.1191 77.5845 77.1603 76.7355 71.7009 57.2064 57.0138 49.1683 46.8269 31.6687 31.1478 29.8273 29.4479 29.0829 28.9388 28.5783 25.0163 22.5098 14.0354 9.0878
6a
(CDCl$_3$, 75 MHz)
f1 (ppm)

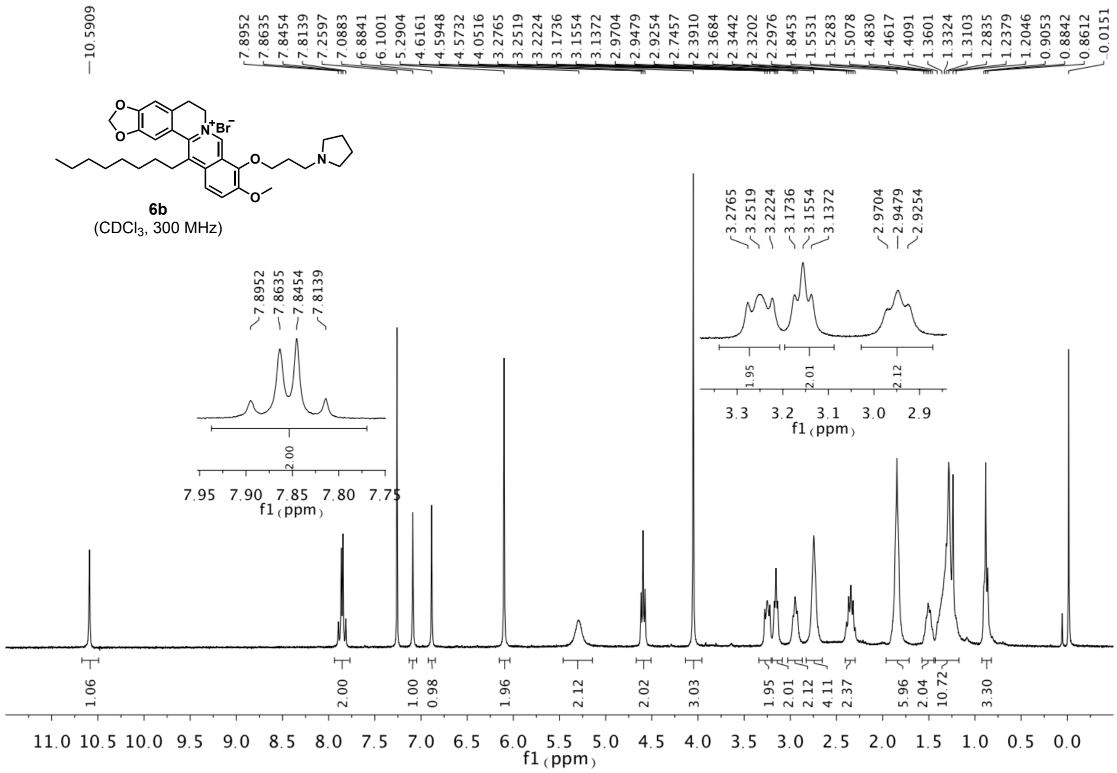
6b
(CDCl$_3$, 300 MHz)
f1 (ppm)

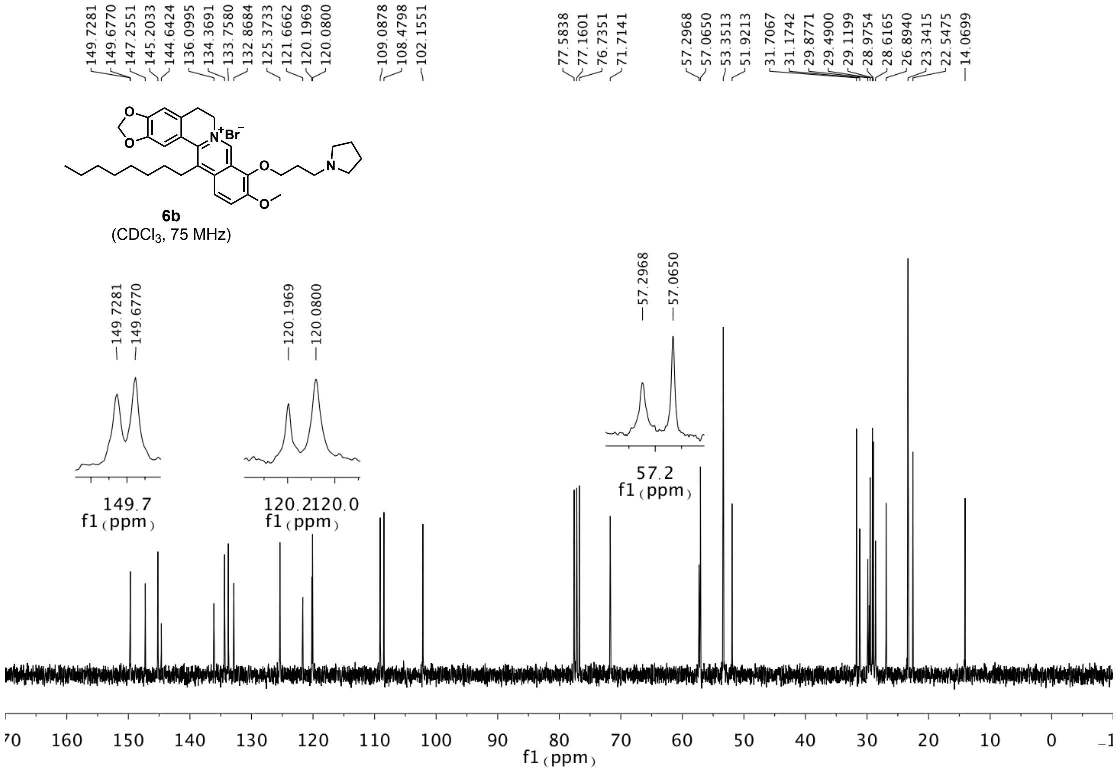
6b
(CDCl$_3$, 75 MHz)
f1 (ppm)

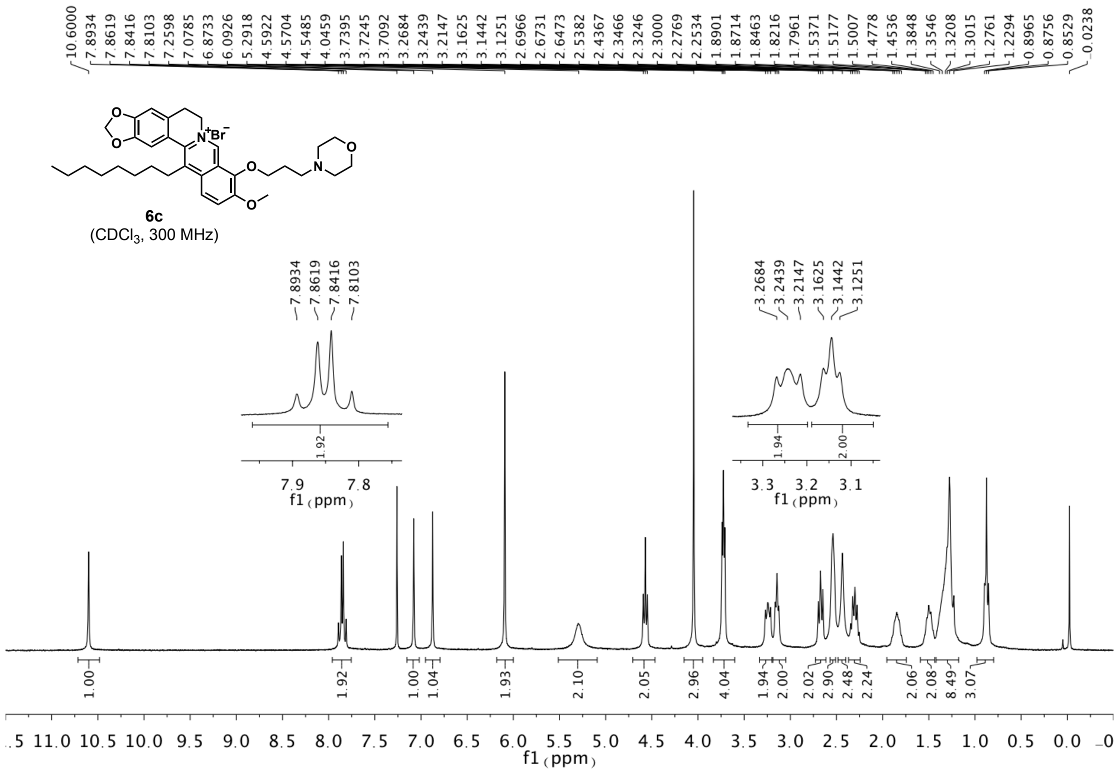
6c
(CDCl$_3$, 300 MHz)
f1 (ppm)

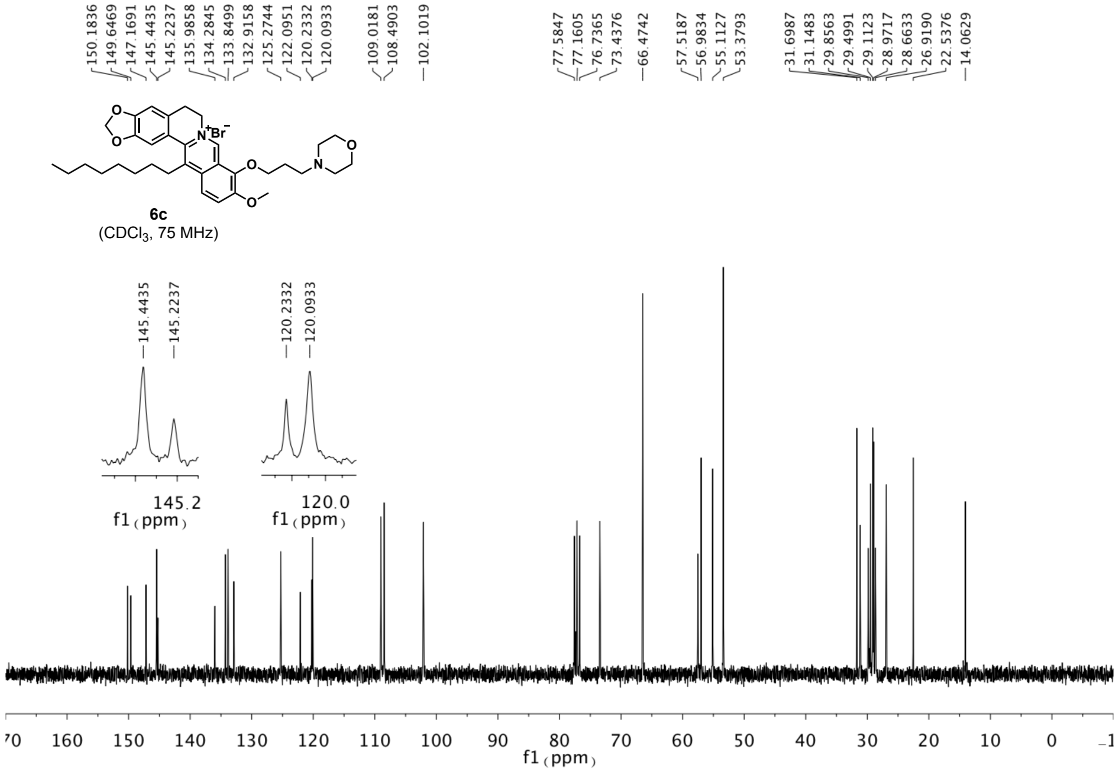
6c
(CDCl$_3$, 75 MHz)
f1 (ppm)

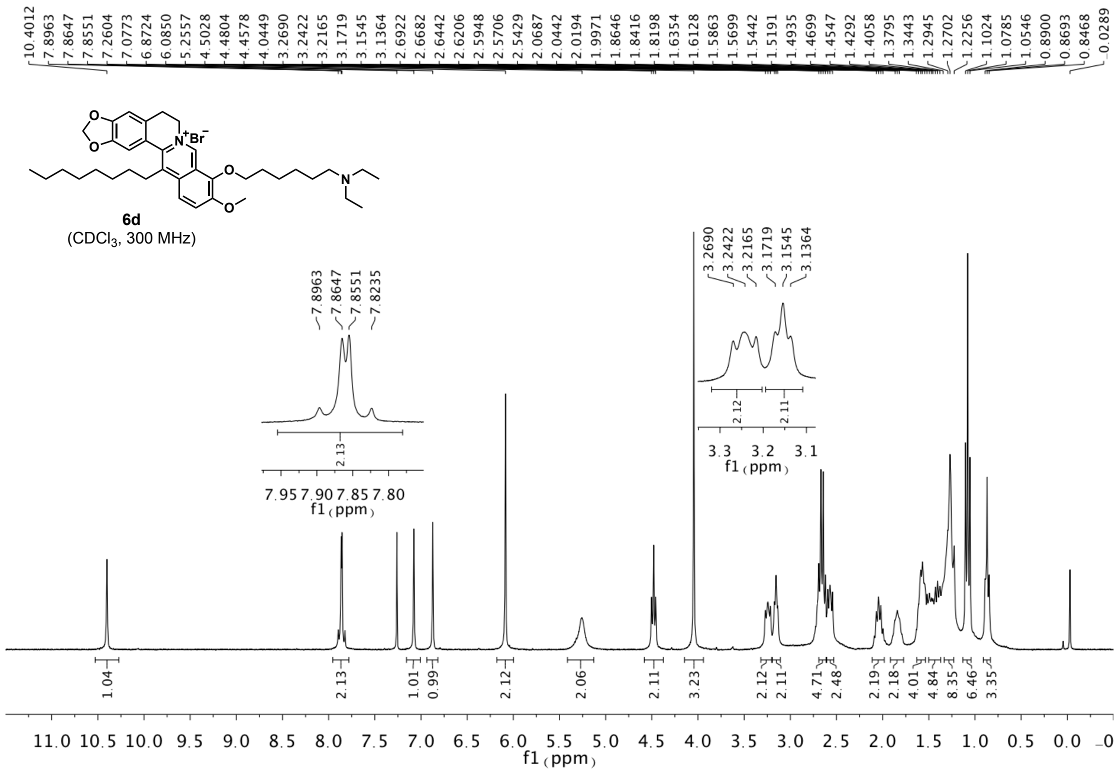

6d
($CDCl_3$, 300 MHz)

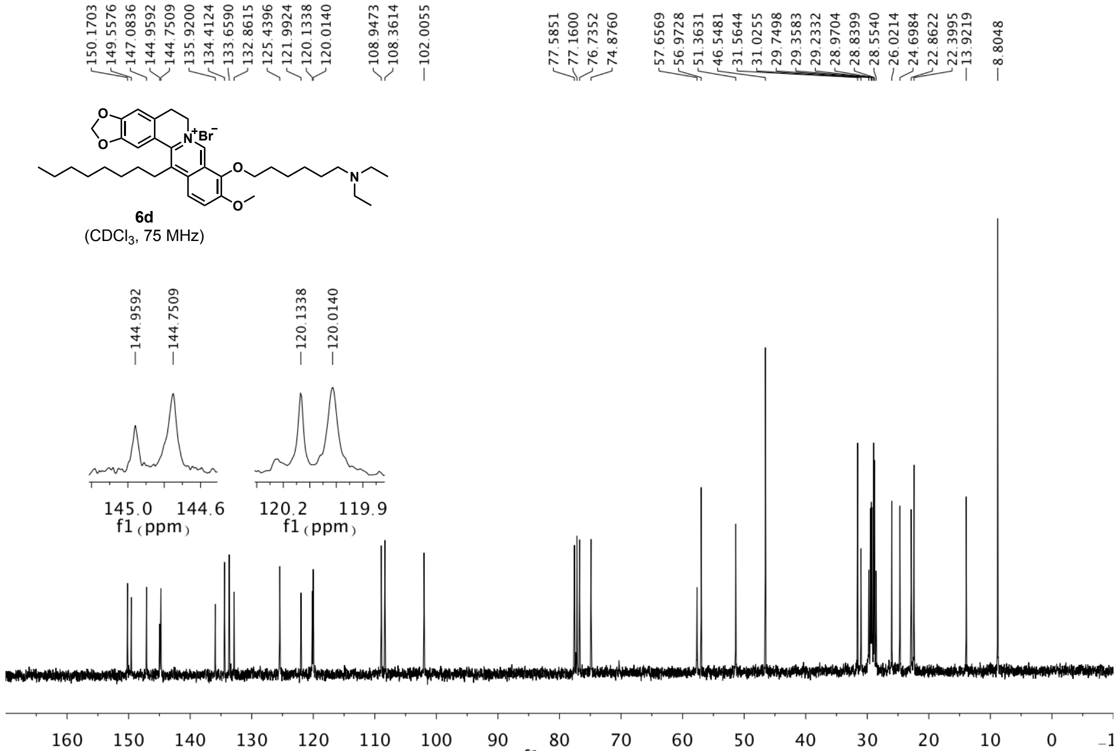

6d
($CDCl_3$, 75 MHz)

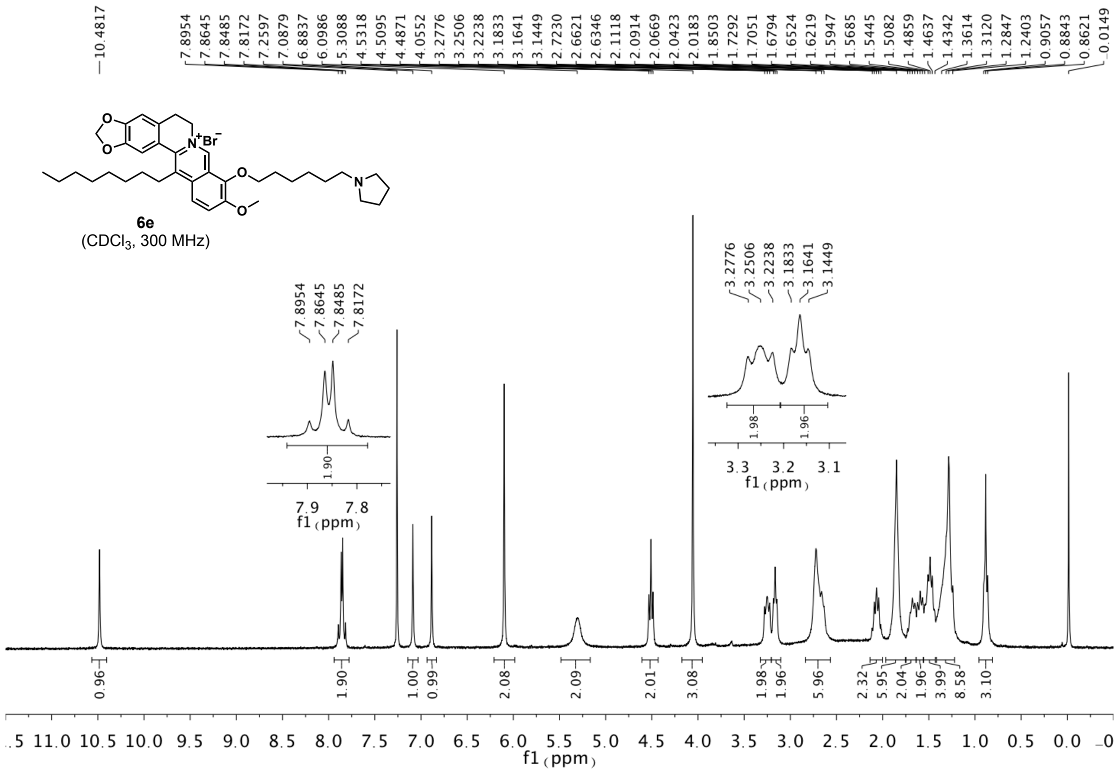

6e
(CDCl$_3$, 300 MHz)

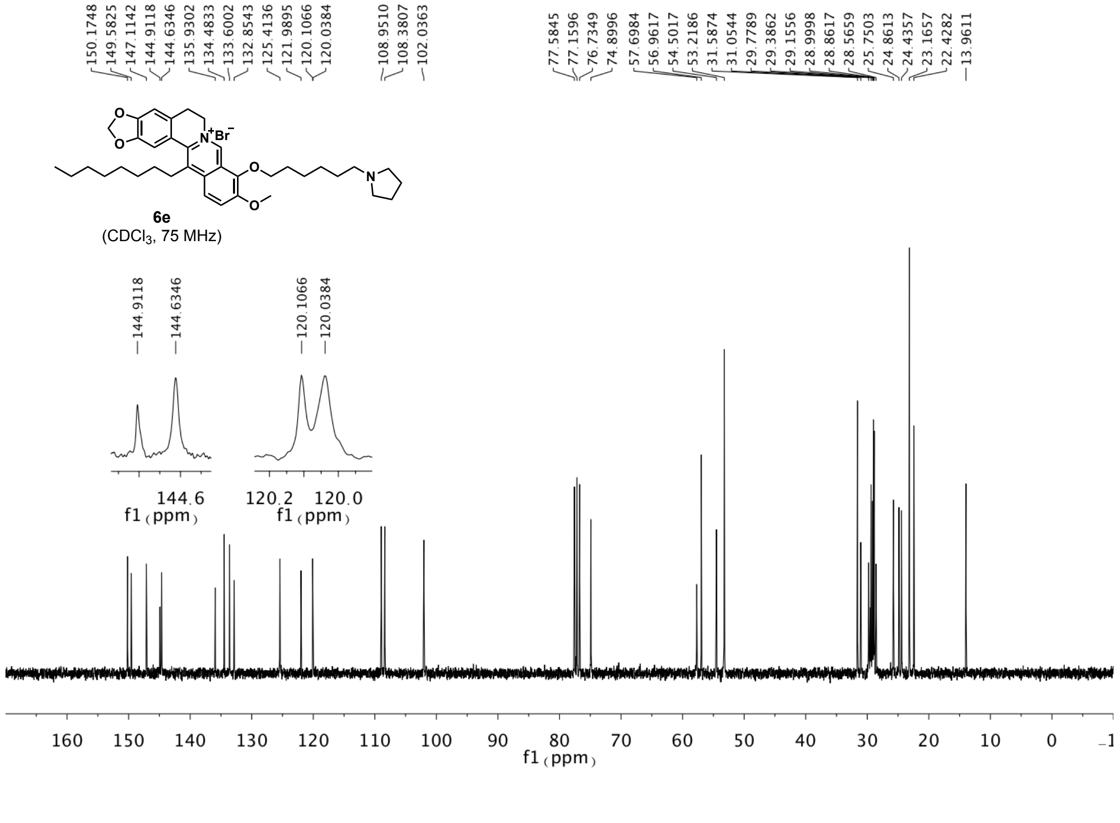

6e
(CDCl$_3$, 75 MHz)

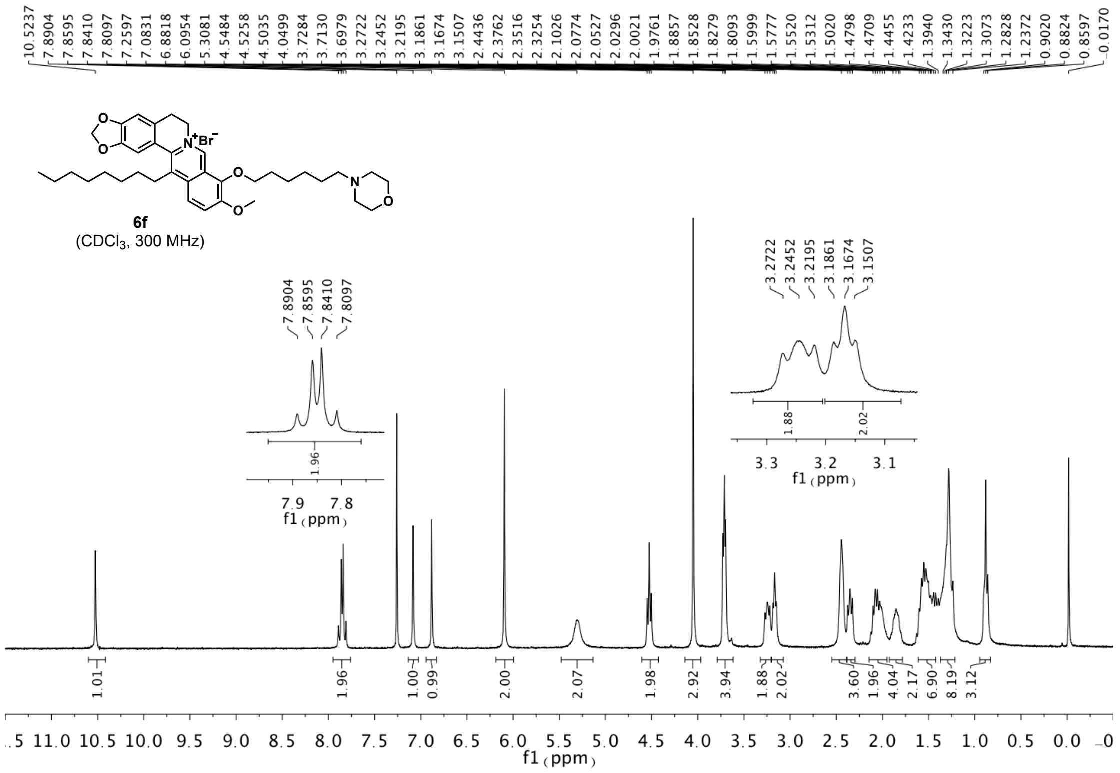

6f
($CDCl_3$, 300 MHz)

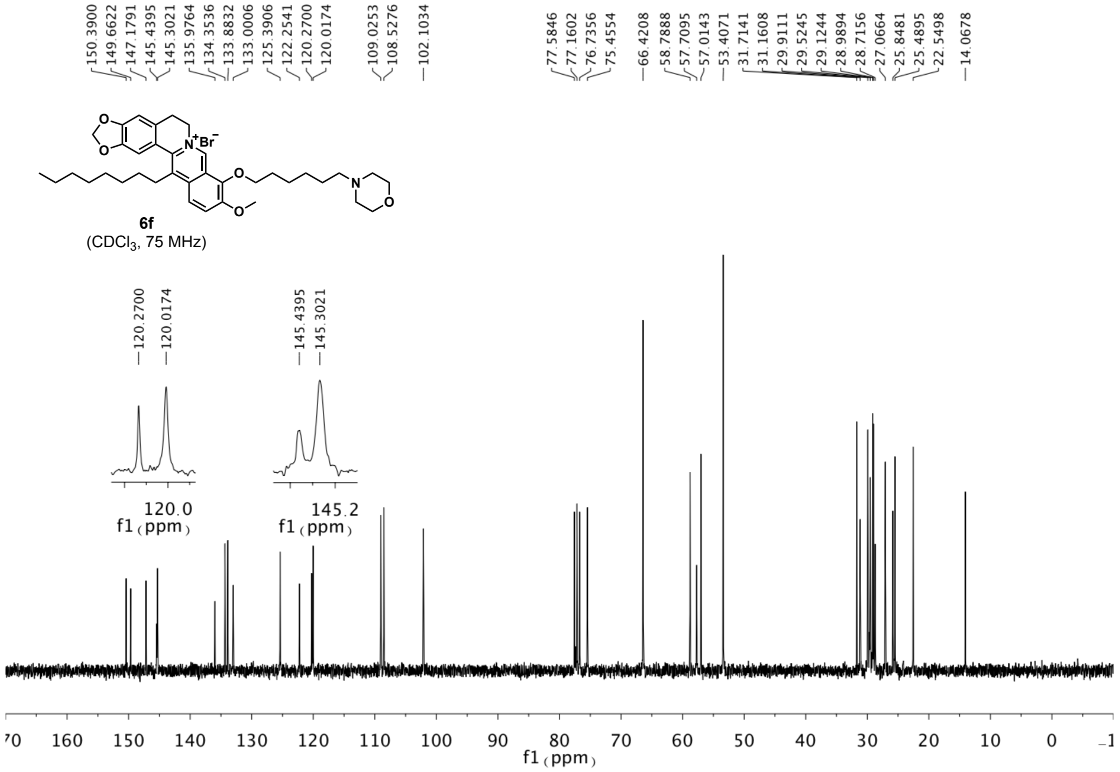

6f
($CDCl_3$, 75 MHz)

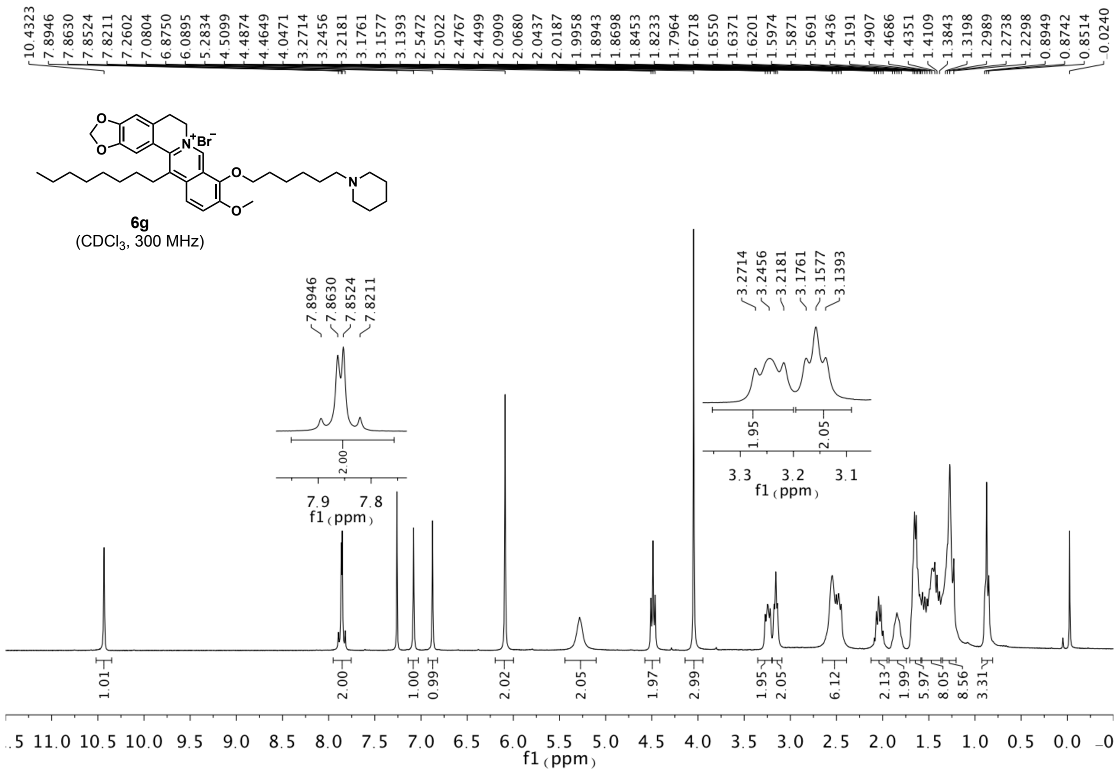
10.4323 7.8946 7.8630 7.8524 7.8211 7.2602 7.0804 6.8750 6.0895 5.2834 4.5099 4.4874 4.4649 4.0471 3.2714 3.2456 3.2181 3.1761 3.1577 3.1393 2.5472 2.5022 2.4767 2.4499 2.0909 2.0680 2.0437 2.0187 1.9958 1.8943 1.8698 1.8453 1.8233 1.7964 1.6718 1.6550 1.6371 1.6201 1.5974 1.5871 1.5691 1.5436 1.5191 1.4907 1.4686 1.4351 1.4109 1.3843 1.3198 1.2989 1.2738 1.2298 0.8949 0.8742 0.8514 0.0240
Br⁻
N
O
6g
(CDCl$_3$, 300 MHz)
7.8946 7.8630 7.8524 7.8211
2.00
7.9 7.8
f1 (ppm)
3.2714 3.2456 3.2181 3.1761 3.1577 3.1393
1.95 2.05
3.3 3.2 3.1
f1 (ppm)
1.01 2.00 1.00 0.99 2.02 2.05 1.97 2.99 1.95 2.05 6.12 2.13 1.99 5.97 8.05 8.56 3.31
11.5 11.0 10.5 10.0 9.5 9.0 8.5 8.0 7.5 7.0 6.5 6.0 5.5 5.0 4.5 4.0 3.5 3.0 2.5 2.0 1.5 1.0 0.5 0.0 -0
f1 (ppm)

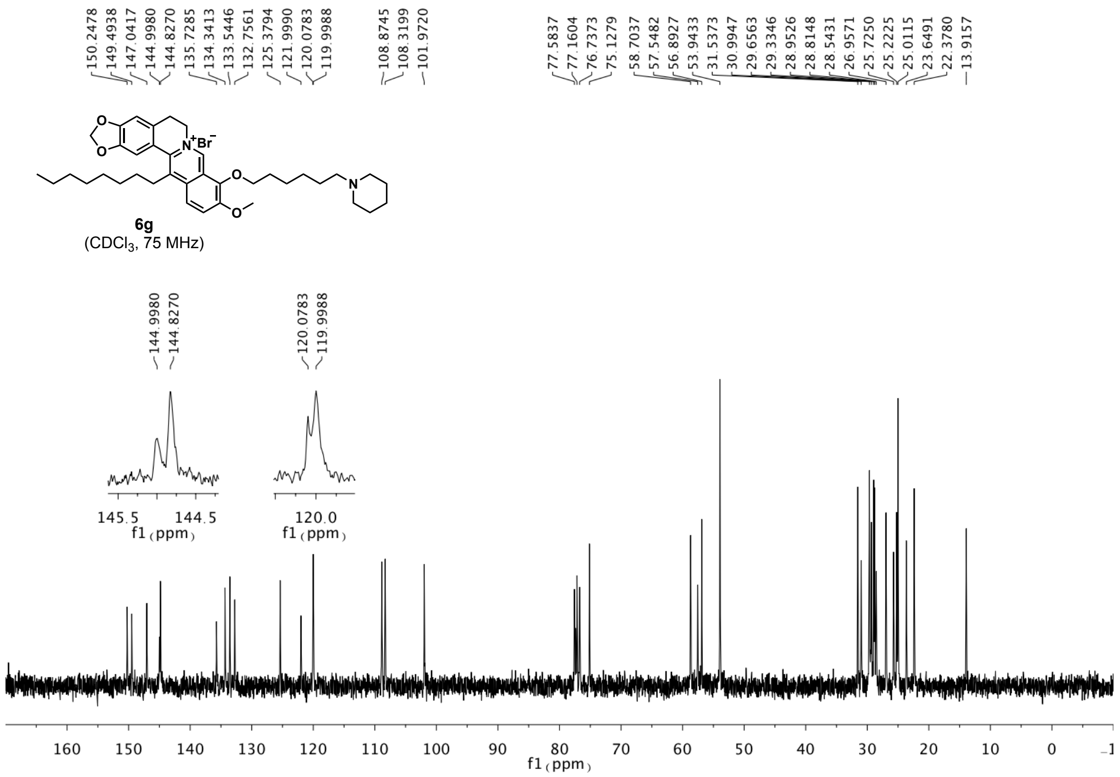
150.2478 149.4938 147.0417 144.9980 144.8270 135.7285 134.3413 133.5446 132.7561 125.3794 121.9990 120.0783 119.9988 108.8745 108.3199 101.9720 77.5837 77.1604 76.7373 75.1279 58.7037 57.5482 56.8927 53.9433 31.5373 30.9947 29.6563 29.3346 28.9526 28.8148 28.5431 26.9571 25.7250 25.2225 25.0115 23.6491 22.3780 13.9157
Br⁻
N
O
6g
(CDCl$_3$, 75 MHz)
144.9980 144.8270
145.5 144.5
f1 (ppm)
120.0783 119.9988
120.0
f1 (ppm)
160 150 140 130 120 110 100 90 80 70 60 50 40 30 20 10 0 -1
f1 (ppm)

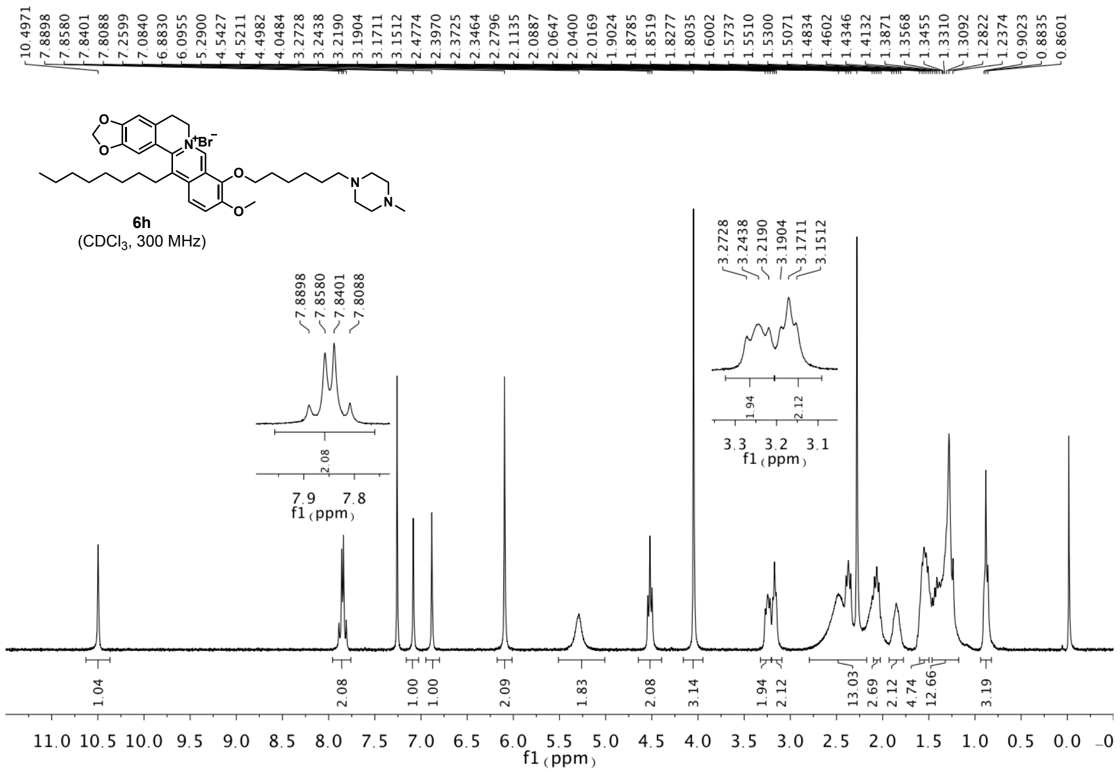
10.4971 7.8898 7.8580 7.8401 7.8088 7.2599 7.0840 6.8830 6.0955 5.2900 4.5427 4.5211 4.4982 4.0484 3.2728 3.2438 3.2190 3.1904 3.1711 3.1512 2.4774 2.3970 2.3725 2.3464 2.2796 2.1135 2.0887 2.0647 2.0400 2.0169 1.9024 1.8785 1.8519 1.8277 1.8035 1.6002 1.5737 1.5510 1.5300 1.5071 1.4834 1.4602 1.4346 1.4132 1.3871 1.3568 1.3455 1.3310 1.3092 1.2822 1.2374 0.9023 0.8835 0.8601
6h
($CDCl_3$, 300 MHz)
f1 (ppm)

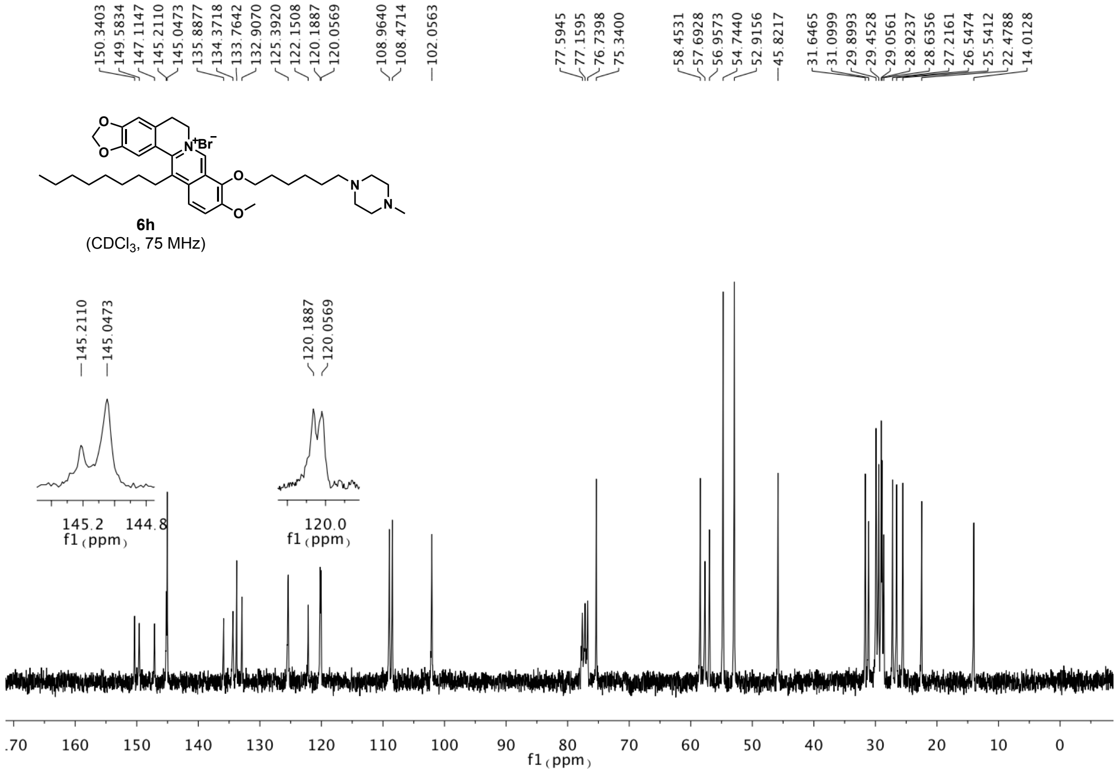
150.3403 149.5834 147.1147 145.2110 145.0473 135.8877 134.3718 133.7642 132.9070 125.3920 122.1508 120.1887 120.0569 108.9640 108.4714 102.0563 77.5945 77.1595 76.7398 75.3400 58.4531 57.6928 56.9573 54.7440 52.9156 45.8217 31.6465 31.0999 29.8993 29.4528 29.0561 28.9237 28.6356 27.2161 26.5474 25.5412 22.4788 14.0128
6h
($CDCl_3$, 75 MHz)
f1 (ppm)

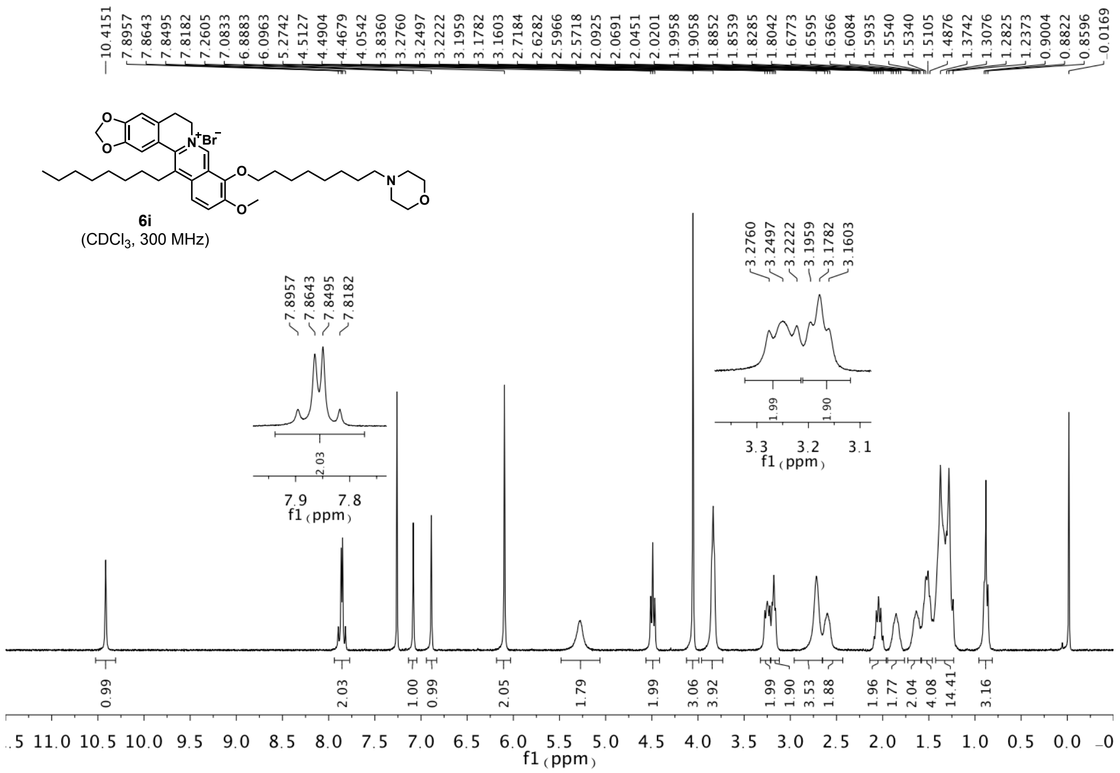
10.4151 7.8957 7.8643 7.8495 7.8182 7.2605 7.0833 6.8883 6.0963 5.2742 4.5127 4.4904 4.4679 4.0542 3.8360 3.2760 3.2497 3.2222 3.1959 3.1782 3.1603 2.7184 2.6282 2.5966 2.5718 2.0925 2.0691 2.0451 2.0201 1.9958 1.9058 1.8852 1.8539 1.8285 1.8042 1.6773 1.6595 1.6366 1.6084 1.5935 1.5540 1.5340 1.5105 1.4876 1.3742 1.3076 1.2825 1.2373 0.9004 0.8822 0.8596 0.0169
6i
(CDCl3, 300 MHz)
0.99 2.03 1.00 0.99 2.05 1.79 1.99 3.06 3.92 1.99 1.90 3.53 1.88 1.96 1.77 2.04 4.08 14.41 3.16
f1 (ppm)

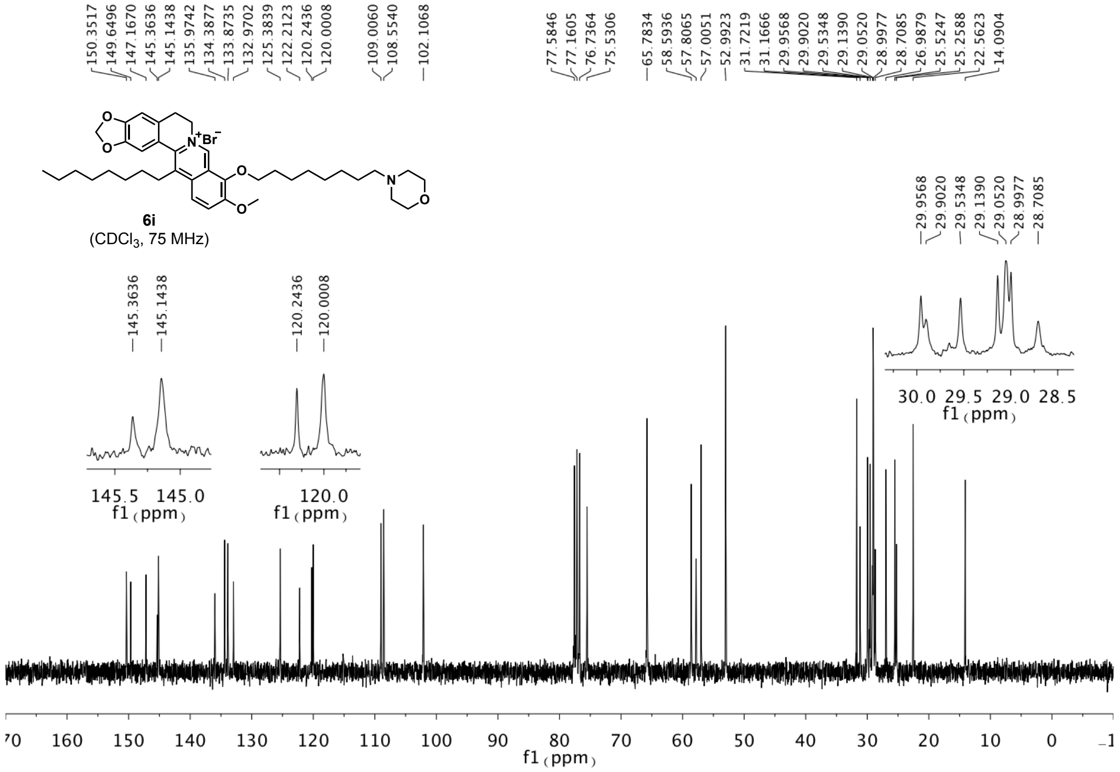
150.3517 149.6496 147.1670 145.3636 145.1438 135.9742 134.3877 133.8735 132.9702 125.3839 122.2123 120.2436 120.0008 109.0060 108.5540 102.1068 77.5846 77.1605 76.7364 75.5306 65.7834 58.5936 57.8065 57.0051 52.9923 31.7219 31.1666 29.9568 29.9020 29.5348 29.1390 29.0520 28.9977 28.7085 26.9879 25.5247 25.2588 22.5623 14.0904
6i
(CDCl3, 75 MHz)
f1 (ppm)

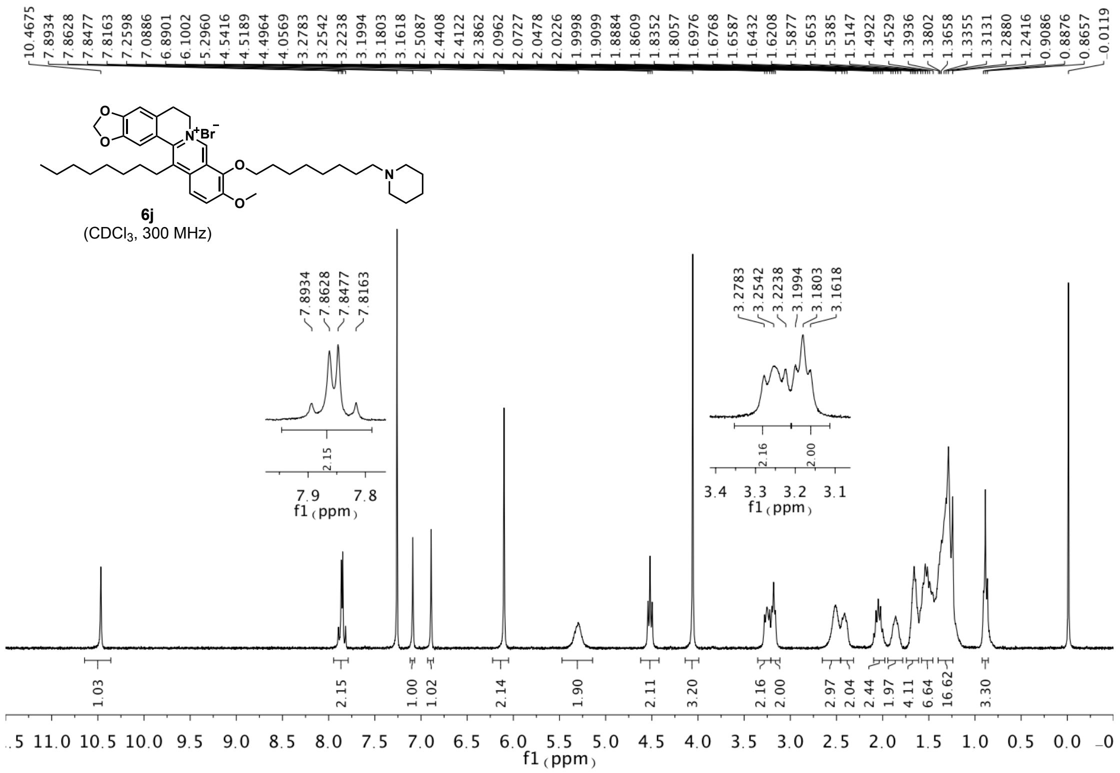
10.4675 7.8934 7.8628 7.8477 7.8163 7.2598 7.0886 6.8901 6.1002 5.2960 4.5416 4.5189 4.4964 4.0569 3.2783 3.2542 3.2238 3.1994 3.1803 3.1618 2.5087 2.4408 2.4122 2.3862 2.0962 2.0727 2.0478 2.0226 1.9998 1.9099 1.8884 1.8609 1.8352 1.8057 1.6976 1.6768 1.6587 1.6432 1.6208 1.5877 1.5653 1.5385 1.5147 1.4922 1.4529 1.3936 1.3802 1.3658 1.3355 1.3131 1.2880 1.2416 0.9086 0.8876 0.8657 0.0119
6j
(CDCl3, 300 MHz)
1.03 2.15 1.00 1.02 2.14 1.90 2.11 3.20 2.16 2.00 2.97 2.04 2.44 1.97 4.11 6.64 16.62 3.30
f1 (ppm)

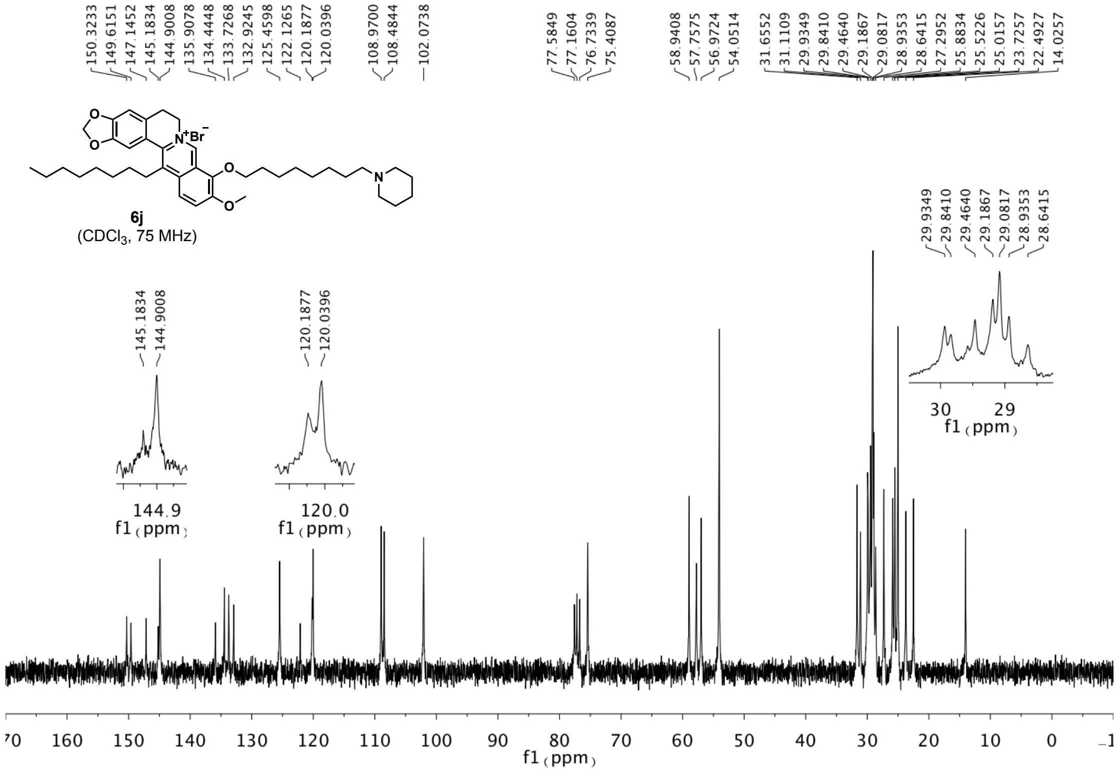
150.3233 149.6151 147.1452 145.1834 144.9008 135.9078 134.4448 133.7268 132.9245 125.4598 122.1265 120.1877 120.0396 108.9700 108.4844 102.0738 77.5849 77.1604 76.7339 75.4087 58.9408 57.7575 56.9724 54.0514 31.6552 31.1109 29.9349 29.8410 29.4640 29.1867 29.0817 28.9353 28.6415 27.2952 25.8834 25.5226 25.0157 23.7257 22.4927 14.0257
6j
(CDCl3, 75 MHz)
f1 (ppm)

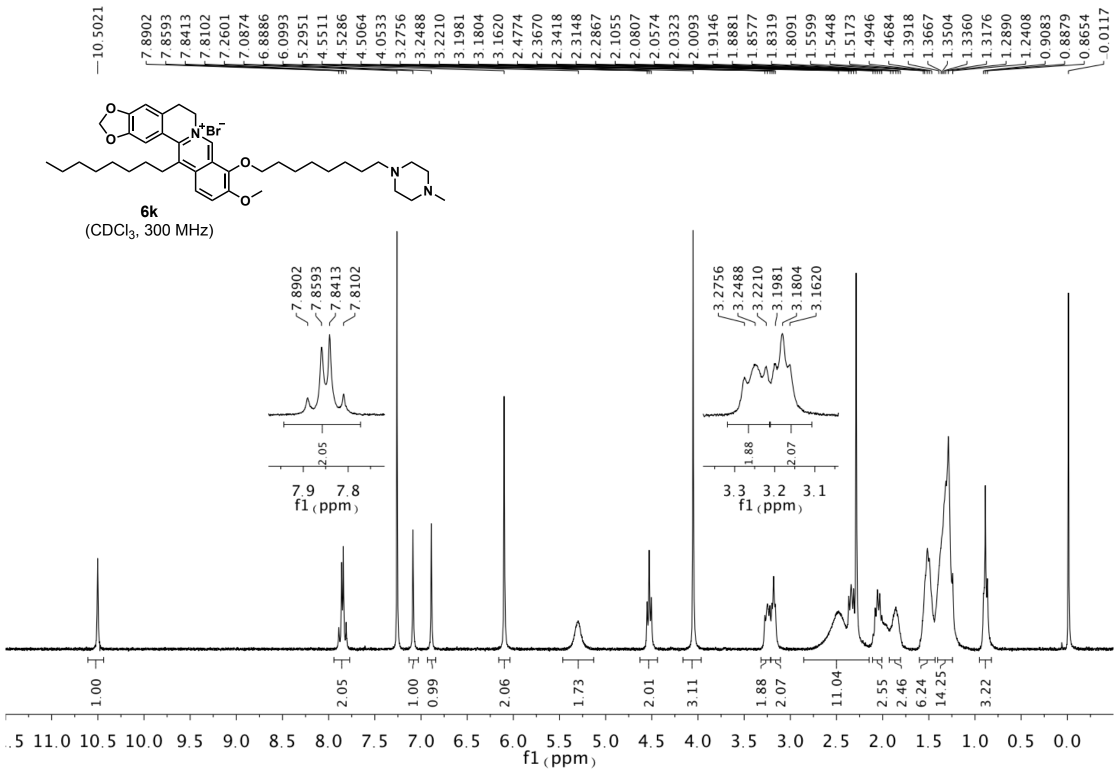
6k
(CDCl3, 300 MHz)
10.5021, 7.8902, 7.8593, 7.8413, 7.8102, 7.2601, 7.0874, 6.8886, 6.0993, 5.2951, 4.5511, 4.5286, 4.5064, 4.0533, 3.2756, 3.2488, 3.2210, 3.1981, 3.1804, 3.1620, 2.4774, 2.3670, 2.3418, 2.3148, 2.2867, 2.1055, 2.0807, 2.0574, 2.0323, 2.0093, 1.9146, 1.8881, 1.8577, 1.8319, 1.8091, 1.5599, 1.5448, 1.5173, 1.4946, 1.4684, 1.3918, 1.3667, 1.3504, 1.3360, 1.3176, 1.2890, 1.2408, 0.9083, 0.8879, 0.8654, 0.0117
f1 (ppm)

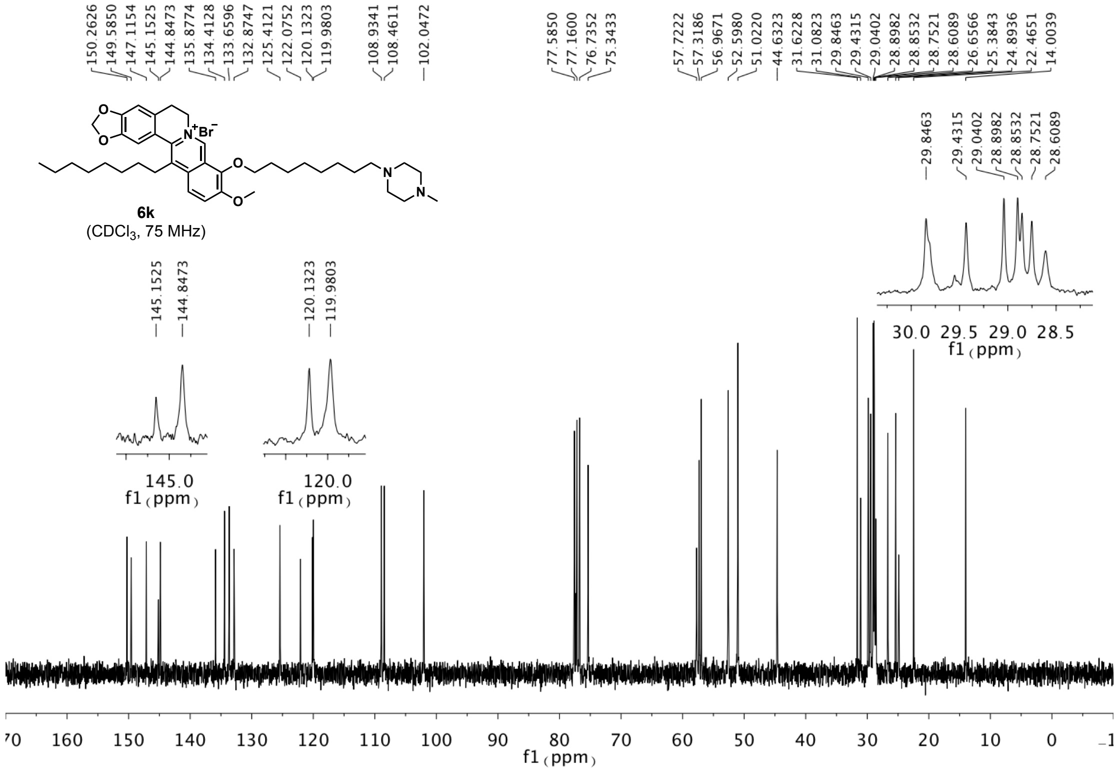
6k
(CDCl3, 75 MHz)
150.2626, 149.5850, 147.1154, 145.1525, 144.8473, 135.8774, 134.4128, 133.6596, 132.8747, 125.4121, 122.0752, 120.1323, 119.9803, 108.9341, 108.4611, 102.0472, 77.5850, 77.1600, 76.7352, 75.3433, 57.7222, 57.3186, 56.9671, 52.5980, 51.0220, 44.6323, 31.6228, 31.0823, 29.8463, 29.4315, 29.0402, 28.8982, 28.8532, 28.7521, 28.6089, 26.6566, 25.3843, 24.8936, 22.4651, 14.0039
f1 (ppm)

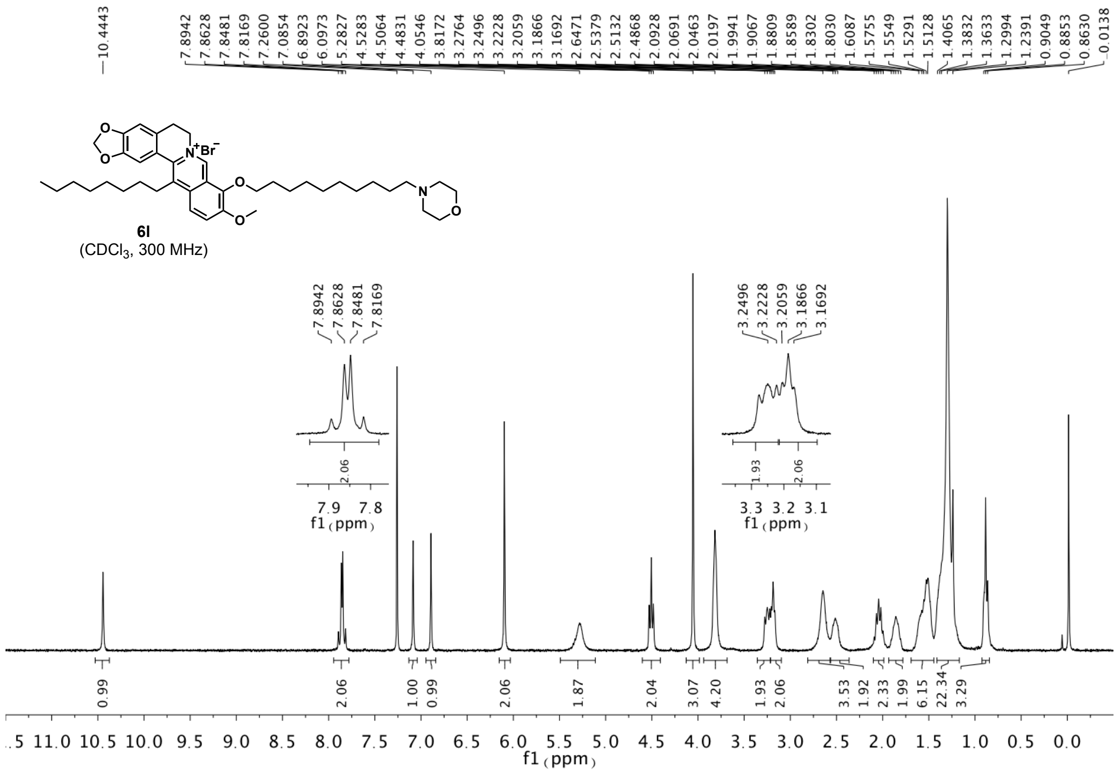
10.4443
7.8942 7.8628 7.8481 7.8169 7.2600 7.0854 6.8923 6.0973 5.2827 4.5283 4.5064 4.4831 4.0546 3.8172 3.2764 3.2496 3.2228 3.2059 3.1866 3.1692 2.6471 2.5379 2.5132 2.4868 2.0928 2.0691 2.0463 2.0197 1.9941 1.9067 1.8809 1.8589 1.8302 1.8030 1.6087 1.5755 1.5549 1.5291 1.5128 1.4065 1.3832 1.3633 1.2994 1.2391 0.9049 0.8853 0.8630 0.0138
6l
(CDCl3, 300 MHz)
7.8942 7.8628 7.8481 7.8169
2.06
7.9 7.8
f1 (ppm)
3.2496 3.2228 3.2059 3.1866 3.1692
1.93 2.06
3.3 3.2 3.1
f1 (ppm)
0.99 2.06 1.00 0.99 2.06 1.87 2.04 3.07 4.20 1.93 2.06 3.53 1.92 2.33 1.99 6.15 22.34 3.29
11.0 10.5 10.0 9.5 9.0 8.5 8.0 7.5 7.0 6.5 6.0 5.5 5.0 4.5 4.0 3.5 3.0 2.5 2.0 1.5 1.0 0.5 0.0
f1 (ppm)

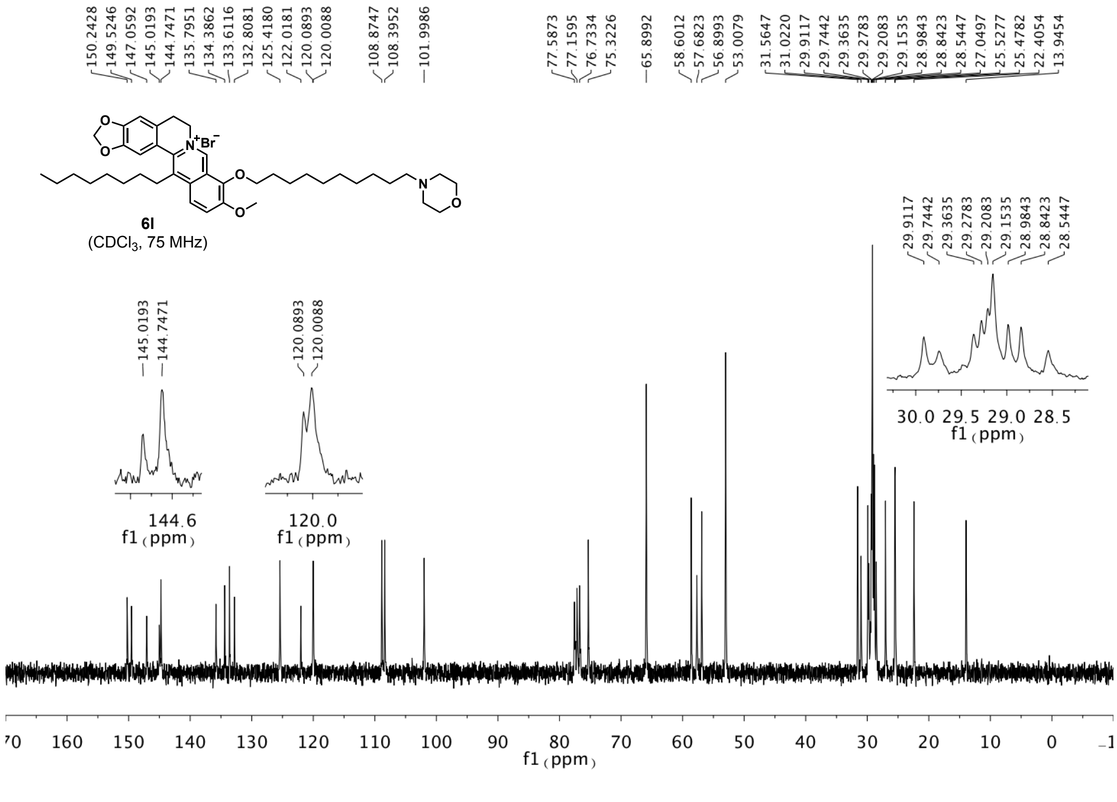
150.2428 149.5246 147.0592 145.0193 144.7471 135.7951 134.3862 133.6116 132.8081 125.4180 122.0181 120.0893 120.0088 108.8747 108.3952 101.9986
77.5873 77.1595 76.7334 75.3226 65.8992 58.6012 57.6823 56.8993 53.0079 31.5647 31.0220 29.9117 29.7442 29.3635 29.2783 29.2083 29.1535 28.9843 28.8423 28.5447 27.0497 25.5277 25.4782 22.4054 13.9454
6l
(CDCl3, 75 MHz)
145.0193 144.7471
144.6
f1 (ppm)
120.0893 120.0088
120.0
f1 (ppm)
29.9117 29.7442 29.3635 29.2783 29.2083 29.1535 28.9843 28.8423 28.5447
30.0 29.5 29.0 28.5
f1 (ppm)
160 150 140 130 120 110 100 90 80 70 60 50 40 30 20 10 0
f1 (ppm)

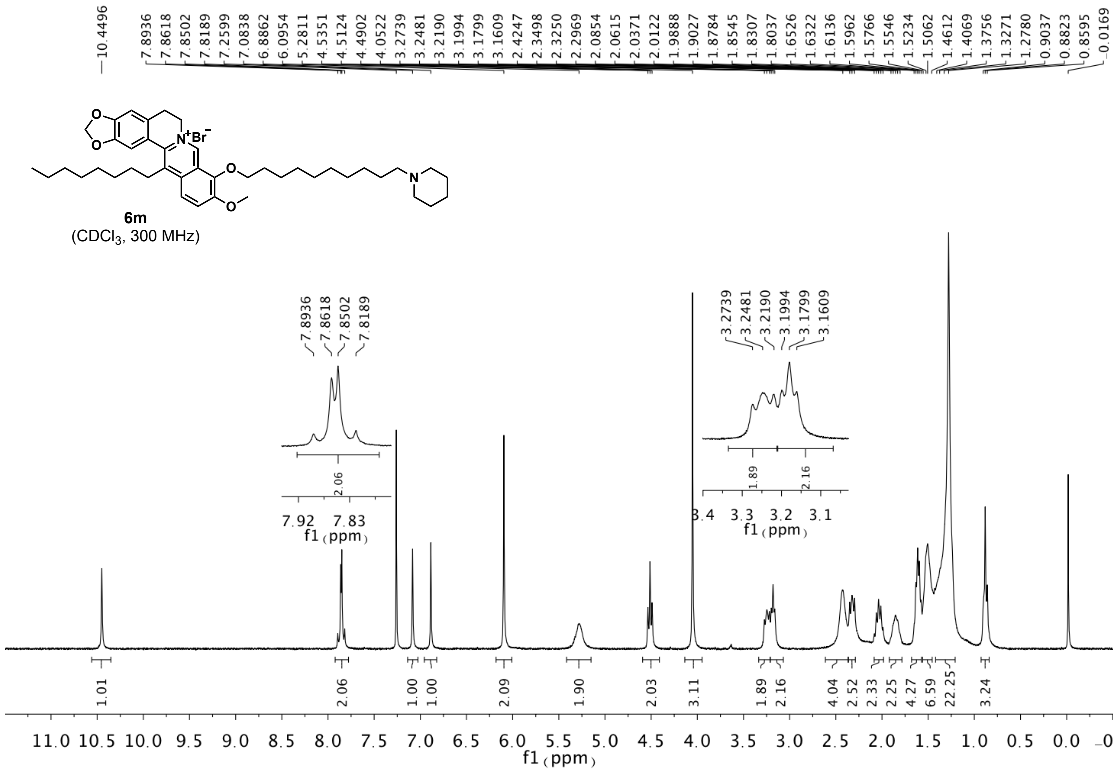
10.4496
7.8936
7.8618
7.8502
7.8189
7.2599
7.0838
6.8862
6.0954
5.2811
4.5351
4.5124
4.4902
4.0522
3.2739
3.2481
3.2190
3.1994
3.1799
3.1609
2.4247
2.3498
2.3250
2.2969
2.0854
2.0615
2.0371
2.0122
1.9888
1.9027
1.8784
1.8545
1.8307
1.8037
1.6526
1.6322
1.6136
1.5962
1.5766
1.5546
1.5234
1.5062
1.4612
1.4069
1.3756
1.3271
1.2780
0.9037
0.8823
0.8595
0.0169
Br
N
O
6m
(CDCl3, 300 MHz)
2.06
7.92 7.83
f1 (ppm)
1.89
2.16
3.4 3.3 3.2 3.1
f1 (ppm)
1.01
2.06
1.00
1.00
2.09
1.90
2.03
3.11
1.89
2.16
4.04
2.52
2.33
2.25
4.27
6.59
22.25
3.24
11.0 10.5 10.0 9.5 9.0 8.5 8.0 7.5 7.0 6.5 6.0 5.5 5.0 4.5 4.0 3.5 3.0 2.5 2.0 1.5 1.0 0.5 0.0 -0
f1 (ppm)

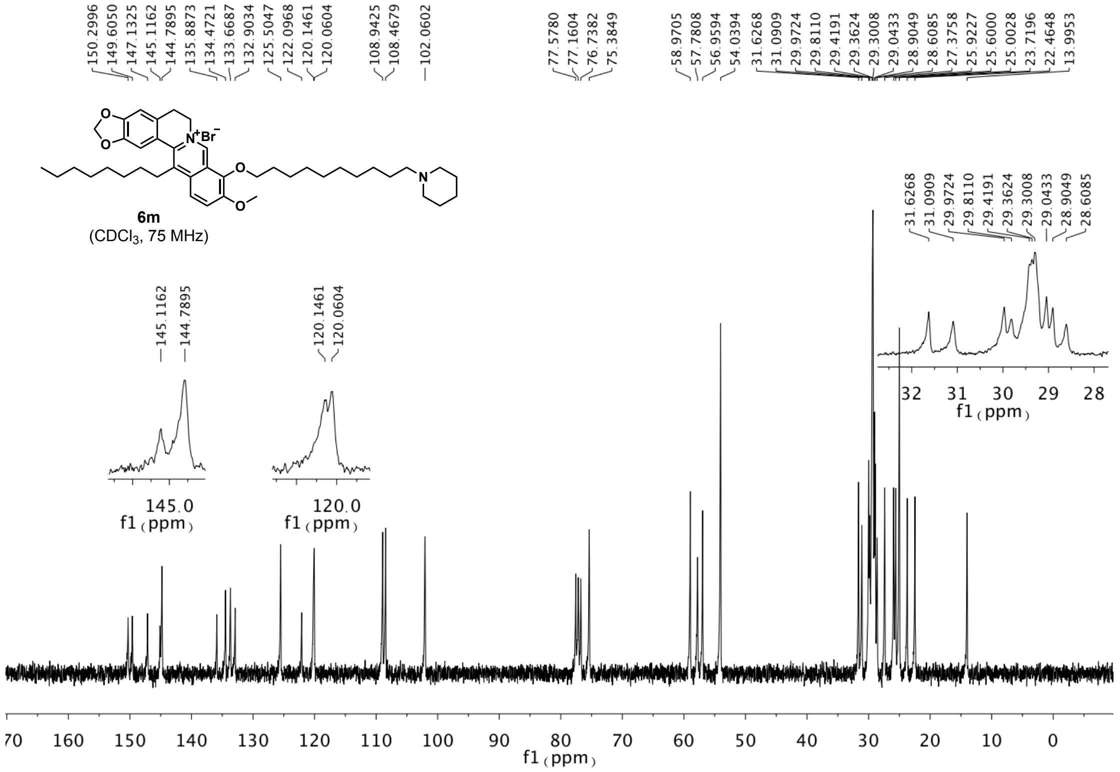
150.2996
149.6050
147.1325
145.1162
144.7895
135.8873
134.4721
133.6687
132.9034
125.5047
122.0968
120.1461
120.0604
108.9425
108.4679
102.0602
77.5780
77.1604
76.7382
75.3849
58.9705
57.7808
56.9594
54.0394
31.6268
31.0909
29.9724
29.8110
29.4191
29.3624
29.3008
29.0433
28.9049
28.6085
27.3758
25.9227
25.6000
25.0028
23.7196
22.4648
13.9953
Br
N
O
6m
(CDCl3, 75 MHz)
145.0
f1 (ppm)
120.0
f1 (ppm)
32 31 30 29 28
f1 (ppm)
70 160 150 140 130 120 110 100 90 80 70 60 50 40 30 20 10 0
f1 (ppm)

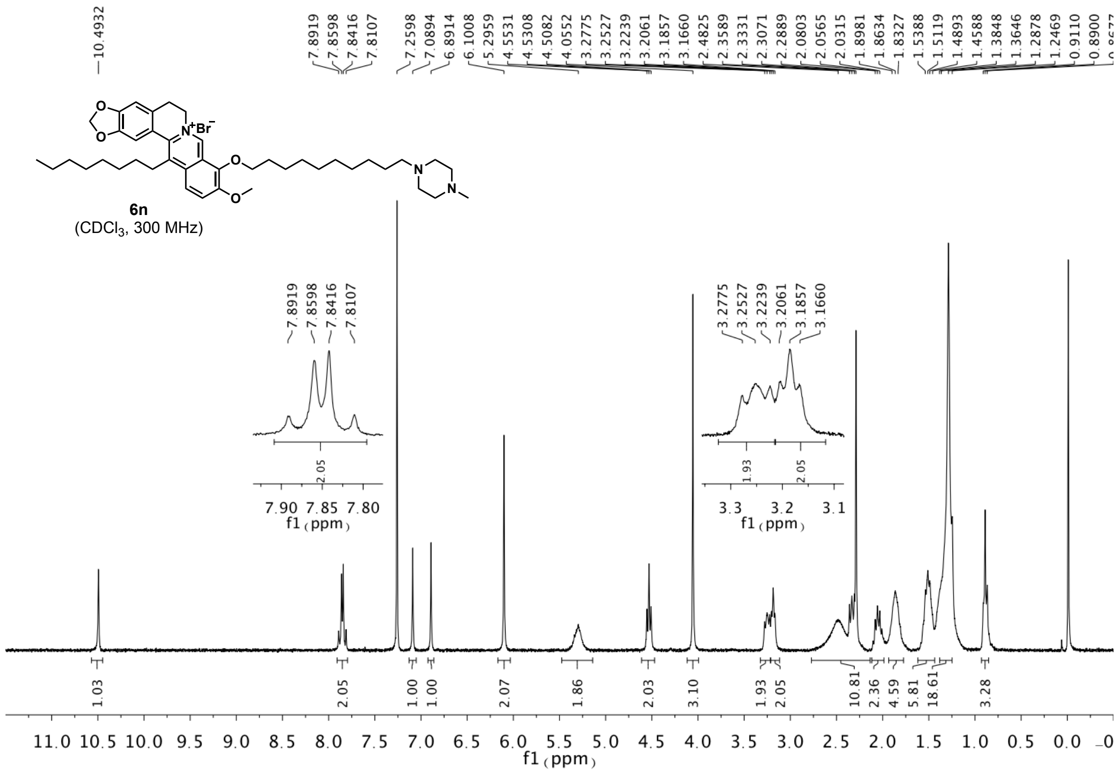
6n
(CDCl$_3$, 300 MHz)
10.4932
7.8919 7.8598 7.8416 7.8107
7.2598 7.0894 6.8914 6.1008 5.2959 4.5531 4.5308 4.5082 4.0552 3.2775 3.2527 3.2239 3.2061 3.1857 3.1660 2.4825 2.3589 2.3331 2.3071 2.2889 2.0803 2.0565 2.0315 1.8981 1.8634 1.8327 1.5388 1.5119 1.4893 1.4588 1.3848 1.3646 1.2878 1.2469 0.9110 0.8900
1.03 2.05 1.00 1.00 2.07 1.86 2.03 3.10 1.93 2.05 10.81 2.36 4.59 5.81 18.61 3.28
f1 (ppm)

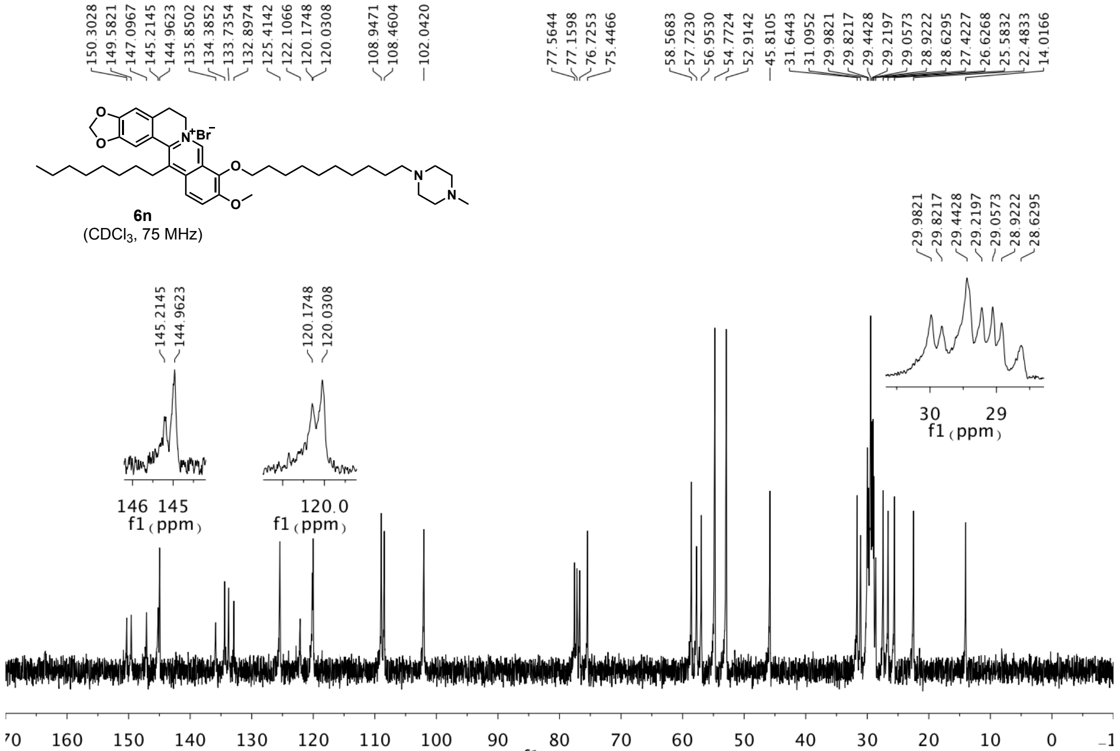
6n
(CDCl$_3$, 75 MHz)
150.3028 149.5821 147.0967 145.2145 144.9623 135.8502 134.3852 133.7354 132.8974 125.4142 122.1066 120.1748 120.0308 108.9471 108.4604 102.0420
77.5644 77.1598 76.7253 75.4466
58.5683 57.7230 56.9530 54.7724 52.9142 45.8105 31.6443 31.0952 29.9821 29.8217 29.4428 29.2197 29.0573 28.9222 28.6295 27.4227 26.6268 25.5832 22.4833 14.0166
f1 (ppm)
